# Global Incidence and mortality of oesophageal cancer and their correlation with socioeconomic indicators temporal patterns and trends in 41 countries

**DOI:** 10.1038/s41598-018-19819-8

**Published:** 2018-03-14

**Authors:** Martin C. S. Wong, Willie Hamilton, David C. Whiteman, Johnny Y. Jiang, Youlin Qiao, Franklin D. H. Fung, Harry H. X. Wang, Philip W. Y. Chiu, Enders K. W. Ng, Justin C. Y. Wu, Jun Yu, Francis K. L. Chan, Joseph J. Y. Sung

**Affiliations:** 1 The JC School of Public Health and Primary Care, Hong Kong, China; 2 The Institute of Digestive Disease, Faculty of Medicine of the Chinese University of Hong Kong (CUHK), Shatin, Hong Kong China; 3 University of Exeter, College House, St Luke’s Campus, Exeter, United Kingdom; 4 QIMR Berghofer Medical Research Institute, Brisbane, Australia; 5 The Chinese Academy of Medical Sciences, Beijing, China; 6 The School of Public Health of the Sun Yat-Sen University, Guangzhou, China; 7 General Practice and Primary Care, Institute of Health and Wellbeing, University of Glasgow, Glasgow, G12 9LX UK; 8 State Key Laboratory of Digestive Disease, CUHK, Shatin, Hong Kong China

## Abstract

Oesophageal cancers (adenocarcinomas [AC] and squamous cell carcinomas [SCC]) are characterized by high incidence/mortality in many countries. We aimed to delineate its global incidence and mortality, and studied whether socioeconomic development and its incidence rate were correlated. The age-standardized rates (ASRs) of incidence and mortality of this medical condition in 2012 for 184 nations from the GLOBOCAN database; national databases capturing incidence rates, and the WHO mortality database were examined. Their correlations with two indicators of socioeconomic development were evaluated. Joinpoint regression analysis was used to generate trends. The ratio between the ASR of AC and SCC was strongly correlated with HDI (r = 0.535 [men]; r = 0.661 [women]) and GDP (r = 0.594 [men]; r = 0.550 [women], both p < 0.001). Countries that reported the largest reduction in incidence in male included Poland (Average Annual Percent Change [AAPC] = −7.1, 95%C.I. = −12,−1.9) and Singapore (AAPC = −5.8, 95%C.I. = −9.5,−1.9), whereas for women the greatest decline was seen in Singapore (AAPC = −12.3, 95%C.I. = −17.3,−6.9) and China (AAPC = −5.6, 95%C.I. = −7.6,−3.4). The Philippines (AAPC = 4.3, 95%C.I. = 2,6.6) and Bulgaria (AAPC = 2.8, 95%C.I. = 0.5,5.1) had a significant mortality increase in men; whilst Columbia (AAPC = −6.1, 95%C.I. = −7.5,−4.6) and Slovenia (AAPC = −4.6, 95%C.I. = −7.9,−1.3) reported mortality decline in women. These findings inform individuals at increased risk for primary prevention.

## Introduction

Globally, oesophageal cancer is one of the most frequently reported malignancies and a leading cause of cancer deaths^1^. In 2008, the disease accounted for around 4 million disability-adjusted life years^2^. The cancer is extremely aggressive and prognosis is often poor^3,4^.

Majority of oesophageal cancers (80%) are reported in more deprived nations. Most of these cancers belong to either of the major histologic types: squamous cell carcinoma (SCC) and adenocarcinoma (AC). Although SCCs historically consist of the majority of all cases of this cancer, in recent years we have observed a rapid rise of AC in western countries^3,5^. The risk factors of AC such as increased age, male sex, obesity, gastro-oesophageal reflux disease, cigarette smoking, and diet low in vegetables and fruit have been widely recognized; whereas for SCC, cigarette smoking, alcohol consumption, caustic injury, poor oral hygiene, ingestion of caustic agents, and nutritional deficiencies are major risk factors^3,6^. As many risk factors of oesophageal cancer could be modified, it should be possible to control this disease by preventive measures^7^. Therefore, it is important to examine its updated rates of incidence and mortality, particularly with respect to temporal trends.

Prior evaluations studying its global trends are based on figures in late 1990s^6–8^; have not taken country-specific socioeconomic development when the trends were compared^6–9^, or compared the incidence/mortality trends in one or few selected years^10^. It remains unknown whether the effect of socioeconomic status on the development of SCC vs. AC is different in different countries. In some western countries surveillance of Barrett’s esophagus is offered, though a tiny fraction of the total burden of oesophageal cancer arises from Barrett’s esophagus.

This study aimed to analyze its global patterns and temporal trends of oesophageal cancer by retrieval of data from national databases. We examined the incidence ratio between AC and SCC and its correlation with socioeconomic development across various countries on a global basis.

## Methodology

### Data retrieval

Estimates of incidence and mortality rates of oesophageal cancer were collected from the GLOBOCAN database for 2012^1^. The disease coding is ICD-10 C15. We used a study approach similar to recent evaluations on prostate, liver, bladder and pancreatic cancer^11–14^. Figures of the Gross Domestic Product (GPD) and Human Development Index (HDI) for each nation were obtained^15^, and incidence/mortality figures from GLOBOCAN of the same year^1^. HDI is determined by income per capita, period of education and life expectancy^15^. We extracted data from various sources, and restricted to those databases with no less than 15 calendar years of incidence/mortality figures. For incidence data, we retrieved country-specific registries based on the Cancer Incidence in Five Continents (CI5) volumes I-X^16^. With an aim to obtain the most updated figures, we used data from the United States^17^ and Europe (European Cancer Observatory and NORDCAN) which are publicly available^18,19^. These databases, including GLOBOCAN, have been used extensively in studies examining the incidence trends of various cancers on a global basis (Supplementary Table 1). Data provided by these databases are comprehensive, and have been recognized as the standards for quality among cancer registries^1,17–22^. Supplementary Table 2 shows the methods by which the data were obtained and their quality. Based on the International Classification of Diseases 10^th^ revision (ICD-10 C15), we categorized incidence data into various groups. Data on mortality were categorized using ICD 9^th^ (150·0) up to 1991 and 10^th^ version (C15) thereafter. The incidence of AC and SCC in 2012 was obtained based on the ICD for Oncology-3 presented in CI5^10,16,23^: ACs: 8140–8141, 8143–8145, 8190–8231, 8260–8263, 8310, 8401, 8480–8490, 8550–8551, 8570–8574, 8576; SCC: 8050–8078 and 8383–8084. To obtain mortality figures, the WHO mortality data series were used. We only included data with quality that reached medium level or above^24^, so that complete and accurate data with wide coverage could be analyzed. The database used death certificates to ascertain causes of death. The ASRs were based on the standard population of the world^25^. More developed nations were defined as European nations, New Zealand, Australia and Northern America as well as Japan, while other countries and regions were considered as less developed nations^1^.

### Study Design

To correlate rates of incidence and mortality vs. the socioeconomic indices (HDI/GDP per capita), a cross-sectional association study was performed. To examine the incidence/mortality trends in various nations based on Average Annual Percent Change (AAPC), we adopted a retrospective review of published age-standardized incidence/mortality rates from international or national data registries by joinpoint regression.

### Statistical Analysis

The ASRs of incidence and mortality, as well as the AC: SCC incidence ratios of each country^10^ were correlated with the two socioeconomic indicators. We used simple linear regression and examined the goodness-of-fit, which were represented by correlation coefficients. We employed regression analysis via joinpoints to evaluate the trends of incidence/mortality^26^. We conducted logarithmic transformation of the incidence/mortality rates and calculated standard errors approximated binomially. We specified a maximum of 3 joinpoints was specified as the analysis option. The intervals of average annual percentage change (AAPC) were evaluated. We calculated the AAPC as an average of APCs by geometric weighting. Weights equal to the length of each segment were assigned for the time interval specified^27^. We compared the magnitude of AAPC with zero which could generate the statistical significance of AAPC, and we defined a trend as stable if the AAPC was insignificant. We considered all p values < 0·05 as reaching statistical significance. This study obtained ethics approval from the Survey and Behavioral Research Ethics Committee of CUHK.

## Results

### Incidence, mortality and histologic subtype ratios of esophageal cancer

In 2012, there were 455,784 new diagnoses of oesophageal cancer and 400,169 mortality related to the cancer (Tables 1**/** and 2). Around 81% of incident cases and 82% of all mortality were reported in underdeveloped nations. Rates of incidence and mortality were greater in relatively less resourced regions than less deprived countries by 1·5 to 1·7-fold in men, and by 3·4 to 4-fold in women. On a global basis, the rates of incidence varied by 20-fold. Among male, the highest rates of incidence were reported in Eastern Asia (with ASR = 16·9 per 100,000), Southern Africa (13·7), Eastern Africa (11·9) and Northern Europe (8·1), and the lowest in Western Africa (0·8), Central America (1·7), Northern Africa (2·4) and Western Asia (2·9) (Table 1). In female individuals, the greatest rates were reported in middle Africa (ASR per 10,000 = 7·8), Western Africa (6·7), Eastern Asia (5·4) and Eastern Africa (4·2), while the lowest in Micronesia/Polynesia (0·2), Southern Europe (0·6), Central America (0·6) and South-Eastern Asia (1·0). The results were shown in Table 2.

**Table 1:** Age standardized incidence/mortality of cancer of oesophagus in various regions (male).

World regions	Population Male (1,000)	Incidence		Mortality		Incidence to mortality ratio
		n	ASR	n	ASR	
**Africa**	549,445	16,062	5.6	14,702	5.3	1.09
Eastern Africa	180,243	9,805	11.9	12,982	6.4	0.76
Middle Africa	69,179	1,202	4.2	8,944	11.2	0.13
Northern Africa	106,147	1,864	2.4	1,109	4.0	1.68
Southern Africa	29,735	2,523	13.7	1,720	2.3	1.47
Western Africa	164,141	668	0.8	2,320	12.8	0.29
**Asia**	2,179,003	240,142	11.4	207,984	9.9	1.15
Eastern Asia	813,296	183,024	16.9	155,262	14.1	1.18
South-Eastern Asia	305,225	9,366	3.6	8,545	3.3	1.10
South-Central Asia	933,786	45,315	6.5	41,922	6.0	1.08
Western Asia	126,697	2,437	2.9	2,255	2.7	1.08
America*	303,514	30,350	5.5	26,996	4.8	1.12
Caribbean	20,951	1,056	4.6	954	4.1	1.10
Central America	82,227	1,141	1.7	1,054	1.6	1.08
South America	200,336	13,286	7.0	10,773	5.6	1.23
North America	173,209	14,867	5.4	14,215	5.0	1.05
**Europe**	355,275	35,100	5.8	30,335	4.9	1.16
Central and Eastern Europe	138,249	11,044	5.6	9,892	5.0	1.12
Northern Europe	49,574	7,618	8.1	6,951	7.2	1.10
Southern Europe	74,900	4,516	3.2	4,091	2.8	1.10
Western Europe	92,553	11,922	6.8	9,401	5.0	1.27
**Oceania**	18,859	1,354	5.2	1,200	4.5	1.13
Australia/New Zealand	13,632	1,251	5.4	1,106	4.7	1.13
Melanesia	4,628	86	3.6	77	3.4	1.12
Micronesia/Polynesia	258	17	3.1	17	3.2	1.00
More developed regions	604,008	67,748	6.4	56,099	5.2	1.21
Less developed regions	2,975,297	255,260	10.1	225,118	9.0	1.13
**World**	3,579,305	323,008	9.0	281,217	7.7	1.15

ASR = Age standardized rate per 100,000 in 2012. Source: GLOBOCAN 2012. Numbers are rounded to the nearest 10 or 100, and may not add up to the total. *America = WHO Americas region (PAHO).

**Table 2:** Age standardized incidence/mortality of cancer of oesophagus in various regions (female).

World regions	Population Female (1,000)	Incidence		Mortality		Incidence to mortality ratio
		n	ASR	n	ASR	
**Africa**	549,608	11,459	3.5	10,542	3.3	1.09
Eastern Africa	182,469	10,211	4.2	9,391	3.9	1.09
Middle Africa	69,644	7,468	7.8	6,860	7.3	1.09
Northern Africa	105,353	681	2.0	630	1.8	1.08
Southern Africa	30,816	1,248	1.5	1,151	1.4	1.08
Western Africa	161,327	1,719	6.7	1,582	6.2	1.09
**Asia**	2,081,150	100,333	4.3	90,735	3.8	1.11
Eastern Asia	777,374	66,899	5.4	59,860	4.5	1.12
South-Eastern Asia	306,008	2,942	1.0	2,659	0.9	1.11
South-Central Asia	881,514	28,498	3.9	26,370	3.6	1.08
Western Asia	116,253	1,994	2.1	1,846	1.9	1.08
America*	310,360	9,640	1.4	8,007	1.1	1.20
Caribbean	21,313	343	1.2	291	1.0	1.18
Central America	83,632	462	0.6	429	0.5	1.08
South America	205,415	4,892	2.0	3,660	1.5	1.34
North America	176,585	3,943	1.1	3,627	1.0	1.09
**Europe**	381,747	10,793	1.2	9,201	0.9	1.17
Central and Eastern Europe	155,701	2,608	0.8	7,427	1.1	0.35
Northern Europe	51,252	3,474	2.7	2,136	0.6	1.63
Southern Europe	78,393	1,119	0.6	3,121	2.3	0.36
Western Europe	96,400	3,592	1.6	1,006	0.5	3.57
**Oceania**	18,746	551	1.7	467	1.4	1.18
Australia/New Zealand	13,715	507	1.7	424	1.3	1.20
Melanesia	4,451	43	1.4	42	1.4	1.02
Micronesia/Polynesia	580	1	0.2	1	0.2	1.00
More developed regions	637,294	18,396	1.2	15,249	0.9	1.21
Less developed regions	2,880,901	114,380	4.1	103,703	3.6	1.10
**World**	3,518,195	132,776	3.1	118,952	2.7	1.12

ASR = Age standardized rate per 100,000 in 2012. Source: GLOBOCAN 2012. Numbers are rounded to the nearest 10 or 100, and may not add up to the total. *America = WHO Americas region (PAHO).

In 2012, the variation of rates of mortality was approximately 10-fold on a global basis. In male, the highest rate of mortality were estimated in the Eastern Asia (14·1), Western Africa (12·8) and middle Africa (11·2); while in female, the highest death rate was observed in these three regions (ASR of mortality = 4·5, 6·2 and 7·3 per 100,000, respectively). The lowest rates of mortality were reported in Central America (1·6), Southern Africa (2·3), and Western Asia (2·7) in men. For women, Central America (0·5) and South-Eastern Asia (0·9) reported the lowest rates of mortality. Regions with the largest incidence: mortality ratio in men included Southern Africa (1·47), Western Europe (1·27), and South America (1·23). In women, the highest ratios were reported for female in Western Europe (3·57), Northern Europe (1·63) and South America (1·34).

Table 3 shows the ratio between the incidence of AC and SCC in all countries where data were available^10^. The AC:SCC ratios were in general higher in male individuals. Countries in Eastern and South-East Asia, as well as sub-Saharan Africa had low AC:SCC ratios. When compared with SCC, many countries in the Northern America (e.g. Canada, the US), Northern Europe and Western Europe such as the Netherlands, UK, and Ireland had larger incidence of AC in men.

**Table 3:** The ratio between incidence of adenocarcinoma and squamous cell carcinoma in all countries.

	Male				Female				AC:SCC ratio (male)	AC:SCC ratio (female)
	AC		SCC		AC		SCC			
	ASR	N	ASR	N	ASR	N	ASR	N		
***Sub-Saharan Africa***										
Angola	0.3	12	5.9	238	0.2	7	3.3	155	0.050	0.045
Benin	0.1	1	1.3	31	0.0	0	0.3	7	0.032	0.000
Botswana	0.8	5	14.4	86	0.2	2	4.0	30	0.058	0.067
Burkina Faso	0.1	3	2.0	51	0.0	2	1.2	58	0.059	0.034
Burundi	1.0	17	17.8	343	0.4	9	7.8	198	0.050	0.045
Cameroon	0.1	3	1.4	77	0.0	1	0.6	40	0.039	0.025
Cape Verde	0.0	0	0.3	1	0.0	0	0.0	0	0.000	0.000
Central African Republic	0.2	2	2.7	32	0.1	1	1.7	26	0.063	0.038
Chad	0.1	3	2.0	54	0.1	2	1.4	44	0.056	0.045
Comoros	0.7	1	11.8	21	0.3	1	6.6	13	0.048	0.077
Congo, Democratic Republic of	0.3	37	5.1	697	0.1	17	2.2	370	0.053	0.046
Congo, Republic of	0.1	1	1.4	14	0.0	0	0.2	3	0.071	0.000
Cote d Ivoire	0.1	6	1.5	93	0.0	1	0.4	25	0.065	0.040
Djibouti	0.2	0	3.6	9	0.2	0	3.8	11	0.000	0.000
Equatorial Guinea	0.1	0	2.1	7	0.0	0	0.3	1	0.000	0.000
Eritrea	0.2	2	3.8	39	0.2	3	4.5	66	0.051	0.045
Ethiopia	0.1	21	1.9	466	0.2	46	4.4	1077	0.045	0.043
France, La Reunion	0.4	2	7.8	36	0.0	0	0.4	3	0.056	0.000
Gabon	0.1	1	2.9	15	0.1	0	1.5	9	0.067	0.000
Ghana	0.0	3	0.8	68	0.0	1	0.2	17	0.044	0.059
Guinea	0.1	1	0.8	19	0.0	0	0.1	4	0.053	0.000
Guinea-Bissau	0.0	0	0.8	3	0.0	0	0.0	0	0.000	0.000
Kenya	1.1	92	19.3	1767	0.7	67	14.3	1481	0.052	0.045
Lesotho	1.1	6	19.8	101	0.5	4	10.6	81	0.059	0.049
Liberia	0.1	1	1.1	9	0.0	0	0.2	3	0.111	0.000
Madagascar	0.6	30	10.6	593	0.2	12	4.3	267	0.051	0.045
Malawi	1.4	51	26.5	1015	0.9	38	19.8	851	0.050	0.045
Mali	0.1	2	1.4	51	0.0	1	0.4	20	0.039	0.050
Mauritania	0.1	0	1.1	10	0.0	0	0.1	1	0.000	0.000
Mauritius	0.3	2	4.2	28	0.1	1	2.1	19	0.071	0.053
Mozambique	0.6	35	11.8	685	0.3	23	6.7	493	0.051	0.047
Namibia	0.1	1	2.2	13	0.0	0	0.5	4	0.077	0.000
Niger	0.1	2	1.1	48	0.0	0	0.2	7	0.042	0.000
Nigeria	0.0	7	0.3	137	0.0	7	0.3	133	0.051	0.053
Rwanda	0.5	11	8.8	208	0.2	5	4.2	119	0.053	0.042
Senegal	0.1	2	1.1	32	0.0	0	0.3	12	0.063	0.000
Sierra Leone	0.1	1	1.2	12	0.0	0	0.3	3	0.083	0.000
Somalia	0.6	12	10.6	238	0.4	10	9.3	233	0.050	0.043
South African Republic	1.0	163	12.9	2121	0.7	162	6.2	1425	0.077	0.114
South Sudan	0.6	16	11.0	298	0.3	10	7.0	210	0.054	0.048
Swaziland	0.5	1	9.1	25	0.1	0	2.9	10	0.040	0.000
Tanzania	0.7	70	12.2	1288	0.3	36	5.8	763	0.054	0.047
The Gambia	0.1	0	1.0	5	0.0	0	0.0	0	0.000	0.000
Togo	0.2	4	3.8	61	0.1	2	1.9	37	0.066	0.054
Uganda	1.3	79	23.3	1518	0.5	36	9.8	726	0.052	0.050
Zambia	0.6	17	10.4	311	0.3	11	7.1	240	0.055	0.046
Zimbabwe	2.6	90	9.0	306	0.9	40	7.1	289	0.294	0.138
***Northern Africa & Western Asia***										
Algeria	0.3	38	0.6	84	0.0	6	0.3	39	0.452	0.154
Armenia	0.7	12	1.5	26	0.1	3	0.5	18	0.462	0.167
Azerbaijan	1.9	78	4.2	171	0.5	26	2.8	160	0.456	0.163
Bahrain	1.5	4	0.7	3	0.3	1	1.0	2	1.333	0.500
Egypt	0.5	164	2.3	736	0.3	102	1.2	439	0.223	0.232
Gaza Strip and West Bank	0.3	10	0.7	22	0.0	2	0.3	13	0.455	0.154
Georgia	0.4	30	1.0	67	0.1	12	0.8	78	0.448	0.154
Iraq	0.9	47	0.7	37	0.2	14	0.6	40	1.270	0.350
Israel	0.4	7	0.8	15	0.1	2	0.5	9	0.467	0.222
Jordan	0.2	2	0.5	5	0.1	1	0.8	3	0.400	0.333
Kuwait	0.3	6	0.6	13	0.1	2	0.5	13	0.462	0.154
Lebanon	0.5	10	1.1	23	0.0	1	0.2	7	0.435	0.143
Libya	0.5	66	1.1	145	0.1	17	0.7	104	0.455	0.163
Morocco	0.6	4	1.2	9	0.2	1	1.0	6	0.444	0.167
Oman	0.6	3	1.2	6	0.4	1	2.5	3	0.500	0.333
Qatar	0.4	39	1.0	82	0.2	11	1.3	93	0.476	0.118
Saudi Arabia	0.7	7	1.6	15	0.1	1	0.7	7	0.467	0.143
Sudan	1.8	169	4.0	373	0.6	66	4.1	434	0.453	0.152
Syrian Arab Republic	0.4	26	0.9	58	0.1	7	0.7	45	0.448	0.156
Tunisia	0.2	9	0.4	20	0.1	4	0.4	24	0.450	0.167
Turkey	1.1	352	3.1	1022	0.2	82	2.8	1059	0.344	0.077
United Arab Emirates	0.5	7	1.2	16	0.4	2	2.2	14	0.438	0.143
Western Sahara	0.1	0	0.3	1	0.0	0	0.0	0	0.000	0.000
Yemen	1.4	63	3.0	137	0.6	34	3.8	223	0.460	0.152
***Central Asia***										
Afghanistan	1.3	84	10.5	712	0.5	33	7.0	490	0.118	0.067
Bangladesh	1.7	901	14.1	7619	0.6	339	8.9	4987	0.118	0.068
Bhutan	0.7	2	5.8	17	0.3	1	4.5	12	0.118	0.083
India	0.4	2174	4.9	24855	0.1	680	2.6	13884	0.087	0.049
Iran, Islamic Republic of	0.9	272	8.2	2626	0.9	253	7.2	2192	0.104	0.115
Kazakhstan	1.7	103	14.0	856	0.5	51	6.2	666	0.120	0.077
Kyrgyzstan	0.7	11	6.1	96	0.2	5	3.2	73	0.115	0.068
Maldives	0.6	1	5.3	6	0.2	0	2.4	3	0.167	0.000
Nepal	0.4	35	3.2	292	0.1	12	1.5	164	0.120	0.073
Pakistan	0.4	257	3.5	2180	0.3	169	4.1	2541	0.118	0.067
Sri Lanka	0.6	71	5.0	592	0.4	50	5.1	688	0.120	0.073
Tajikistan	2.1	36	17.5	304	0.7	15	10.0	212	0.118	0.071
Turkmenistan	2.6	40	21.3	341	1.1	22	15.2	312	0.117	0.071
Uzbekistan	0.8	67	6.4	557	0.3	36	4.7	515	0.120	0.070
***Eastern/South-East Asia***										
Brunei	0.0	0	0.4	1	0.1	0	1.1	1	0.000	0.000
Cambodia	0.2	7	3.9	152	0.1	3	1.2	72	0.046	0.042
China	0.8	7009	17.5	151146	0.3	2489	6.3	59639	0.046	0.042
Indonesia	0.1	67	1.4	1427	0.0	33	0.6	637	0.047	0.052
Japan	0.4	633	10.5	15640	0.1	164	1.6	2897	0.040	0.057
Korea, Democratic Republic of	0.4	54	9.0	1157	0.1	21	1.8	366	0.047	0.057
Korea, Republic of	0.2	78	5.7	1925	0.0	21	0.4	166	0.041	0.127
Lao PDR	0.0	1	0.9	16	0.0	0	0.3	8	0.063	0.000
Malaysia	1.0	121	1.5	177	0.5	62	1.0	123	0.684	0.504
Mongolia	1.0	8	19.9	156	0.8	8	13.9	140	0.051	0.057
Myanmar	0.5	99	10.8	2312	0.2	42	3.9	943	0.043	0.045
Philippines	0.6	154	1.2	345	0.1	50	0.4	143	0.446	0.350
Singapore	0.3	12	2.5	92	0.1	3	0.5	26	0.130	0.115
Thailand	0.3	114	4.1	1743	0.1	39	0.8	391	0.065	0.100
Timor-Leste	0.1	0	2.4	8	0.1	0	0.8	2	0.000	0.000
***Central and Southern America & Caribbean***										
Argentina	2.0	473	4.2	1020	0.4	163	1.7	603	0.464	0.270
Bahamas	0.6	1	2.1	4	0.1	0	0.4	1	0.250	0.000
Barbados	0.8	2	2.5	5	0.2	1	0.5	2	0.400	0.500
Belize	0.9	1	3.2	3	0.3	0	1.2	2	0.333	0.000
Bolivia	0.3	10	1.0	33	0.1	3	0.3	11	0.303	0.273
Brazil	2.0	1873	8.1	7804	0.6	706	2.1	2469	0.240	0.286
Chile	0.7	78	4.1	441	0.3	39	1.5	230	0.177	0.170
Colombia	1.1	216	1.9	357	0.2	53	0.9	220	0.605	0.241
Costa Rica	0.8	18	1.2	28	0.2	4	0.4	13	0.643	0.308
Cuba	1.7	147	6.0	510	0.4	39	1.3	132	0.288	0.295
Dominican Republic	0.4	19	1.3	63	0.3	12	0.9	40	0.302	0.300
Ecuador	0.3	23	1.1	78	0.2	16	0.3	24	0.295	0.667
El Salvador	0.5	15	1.6	44	0.1	6	0.5	21	0.341	0.286
France, Guadeloupe	1.3	4	4.5	15	0.2	1	0.7	2	0.267	0.500
France, Martinique	1.0	3	3.5	11	0.1	1	0.4	2	0.273	0.500
French Guyana	1.4	1	4.8	5	0.0	0	0.0	0	0.200	0.000
Guatemala	0.5	22	1.6	72	0.3	13	0.9	45	0.306	0.289
Guyana	0.6	1	1.9	5	0.1	0	0.4	2	0.200	0.000
Haiti	0.5	17	1.8	62	0.3	12	1.0	39	0.274	0.308
Honduras	0.5	11	1.5	36	0.1	4	0.4	13	0.306	0.308
Jamaica	0.9	13	2.9	40	0.2	3	0.6	10	0.325	0.300
Mexico	0.4	190	1.2	623	0.1	74	0.4	247	0.305	0.300
Nicaragua	0.4	7	1.4	23	0.0	0	0.1	2	0.304	0.000
Panama	0.6	10	1.8	31	0.2	3	0.5	11	0.323	0.273
Paraguay	1.0	26	3.5	89	0.3	8	1.0	27	0.292	0.296
Peru	0.5	57	1.4	180	0.1	22	0.5	74	0.317	0.297
Puerto Rico	0.9	25	2.4	64	0.3	10	0.5	23	0.391	0.435
Suriname	0.3	1	0.9	2	0.1	0	0.5	2	0.500	0.000
Trinidad and Tobago	0.5	3	1.7	11	0.0	0	0.0	0	0.273	0.000
Uruguay	2.1	51	6.6	157	0.6	26	2.1	78	0.325	0.333
Venezuela	0.5	57	1.5	192	0.1	19	0.4	65	0.297	0.292
***Northern America***										
Canada	3.0	898	1.5	462	0.4	158	0.7	269	1.944	0.587
United States of America	3.6	8785	1.8	4334	0.4	1309	0.7	2090	2.027	0.626
***Eastern Europe***										
Belarus	0.8	50	5.9	356	0.1	12	0.3	29	0.140	0.414
Bulgaria	0.9	55	2.1	125	0.1	13	0.3	26	0.440	0.500
Czech Republic	2.2	188	3.4	285	0.3	36	0.7	73	0.660	0.493
Hungary	1.3	94	5.6	407	0.2	25	0.7	70	0.231	0.357
Poland	0.5	148	3.4	989	0.1	43	0.7	311	0.150	0.138
Republic of Moldova	0.7	15	2.7	62	0.1	2	0.1	4	0.242	0.500
Romania	0.8	121	3.5	527	0.1	31	0.4	80	0.230	0.388
Russian Federation	0.8	699	5.6	4953	0.1	232	0.7	1268	0.141	0.183
Slovakia	0.8	29	5.6	209	0.2	8	0.7	34	0.139	0.235
Ukraine	0.9	271	4.5	1339	0.2	91	0.3	165	0.202	0.552
***Northern and Western Europe***										
Austria	2.0	150	3	216	0.2	24	0.5	50	0.694	0.480
Belgium	3.5	351	3.9	359	0.6	80	1.4	159	0.978	0.503
Denmark	3.1	169	2.4	122	0.8	50	1.5	91	1.385	0.549
Estonia	0.4	4	4	38	0.1	1	0.5	10	0.105	0.100
Finland	1.8	94	1.8	96	0.3	25	0.8	60	0.979	0.417
France (metropolitan)	1.6	886	4.4	2331	0.3	264	1.4	873	0.380	0.302
Germany	2.2	1789	4.7	3659	0.3	334	1.1	1050	0.489	0.318
Iceland	3.9	10	2.7	7	0.6	2	0.8	3	1.429	0.667
Ireland	5.4	183	2.9	95	1	44	2.2	94	1.926	0.468
Latvia	0.9	15	6.2	97	0.2	6	0.9	24	0.155	0.250
Lithuania	1.1	25	6.3	139	0.2	7	0.6	24	0.180	0.292
Luxembourg	3.4	13	2.9	11	0.6	3	1.1	6	1.182	0.500
Norway	2.3	102	1.5	65	0.4	25	0.7	35	1.569	0.714
Sweden	2.1	198	1.5	141	0.4	44	0.7	78	1.404	0.564
Switzerland	2.6	197	3.3	237	0.5	46	1.4	111	0.831	0.414
The Netherlands	7.1	1102	2.8	433	1.2	237	1.5	276	2.545	0.859
United Kingdom	7.2	4351	2.5	1510	1.4	1120	2	1617	2.881	0.693
***Southern Europe***										
Albania	0.4	9	1.3	26	0.1	3	0.5	12	0.346	0.250
Bosnia Herzegovina	0.4	13	1.3	38	0.1	5	0.5	16	0.342	0.313
Croatia	0.9	32	4.5	168	0.1	9	0.5	33	0.190	0.273
Cyprus	0.8	6	0.6	5	0.2	2	0.3	3	1.200	0.667
Macedonia	0.3	4	0.9	14	0.1	1	0.2	3	0.286	0.333
Greece	0.4	44	1.2	129	0.1	10	0.2	31	0.341	0.323
Italy	0.6	404	1.5	919	0.1	93	0.4	370	0.440	0.251
Malta	2.3	9	1.3	5	0.1	1	0.6	5	1.800	0.200
Montenegro	0.6	3	2.2	10	0.2	1	0.7	3	0.300	0.333
Portugal	1.3	121	4.7	405	0.1	17	0.4	57	0.299	0.298
Serbia	0.6	44	3.4	252	0.1	12	0.7	68	0.175	0.176
Slovenia	0.7	12	3	54	0.1	3	0.5	14	0.222	0.214
Spain	1.3	527	3.1	1209	0.2	93	0.5	234	0.436	0.397
***Oceania***										
Australia	3.4	657	1.9	365	0.5	119	1.1	285	1.800	0.418
Fiji	0.4	2	2.6	10	0.0	0	0	0	0.200	0.000
French Polynesia	0.7	1	4.8	7	0.0	0	0	0	0.143	0.000
Guam	0.4	0	2.5	3	0.0	0	0	0	0.000	0.000
New Caledonia	0.7	1	4.7	7	0.0	0	0	0	0.143	0.000
New Zealand	4.0	150	1.5	57	0.6	27	1.2	60	2.632	0.450
Papua New Guinea	0.5	8	3.5	56	0.3	7	1.6	36	0.143	0.194
Samoa	0.0	0	0	0	0.2	0	1	1	0.000	0.000
Solomon Islands	0.0	0	0.3	1	0.0	0	0	0	0.000	0.000
Vanuatu	0.0	0	0	0	0.0	0	0	0	0.000	0.000

AC: adenocarcinoma; ASR: age-standardized rate; SCC: squamous cell carcinoma.

### Correlation: rates of incidence/mortality vs. HDI and GDP per capita

**Figure 1:**
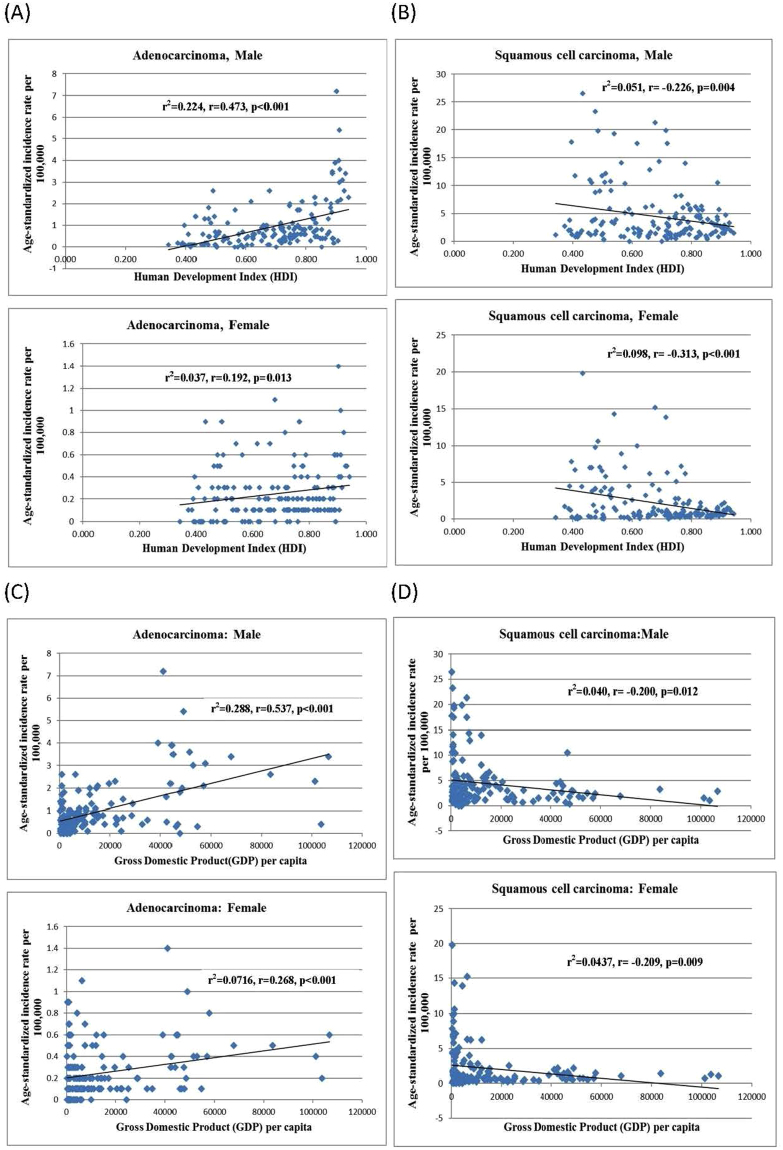
(**A**) Relationship between incidence of oesophageal adenocarcinoma and Human Development Index in male (upper panel) and female (lower panel). (**B**) Relationship between age-standardised incidence of oesophageal squamous cell carcinoma and Human Development Index in male (upper panel) and female (lower panel). (**C**) Relationship between age-standardised incidence of oesophageal adenocarcinoma and Gross Domestic Product per capita in male (upper panel) and female (lower panel). (**D**) Relationship between age-standardised incidence of oesophageal squamous cell carcinoma and Gross Domestic Product per capita in male (upper panel) and female (lower panel).

**Figure 2:**
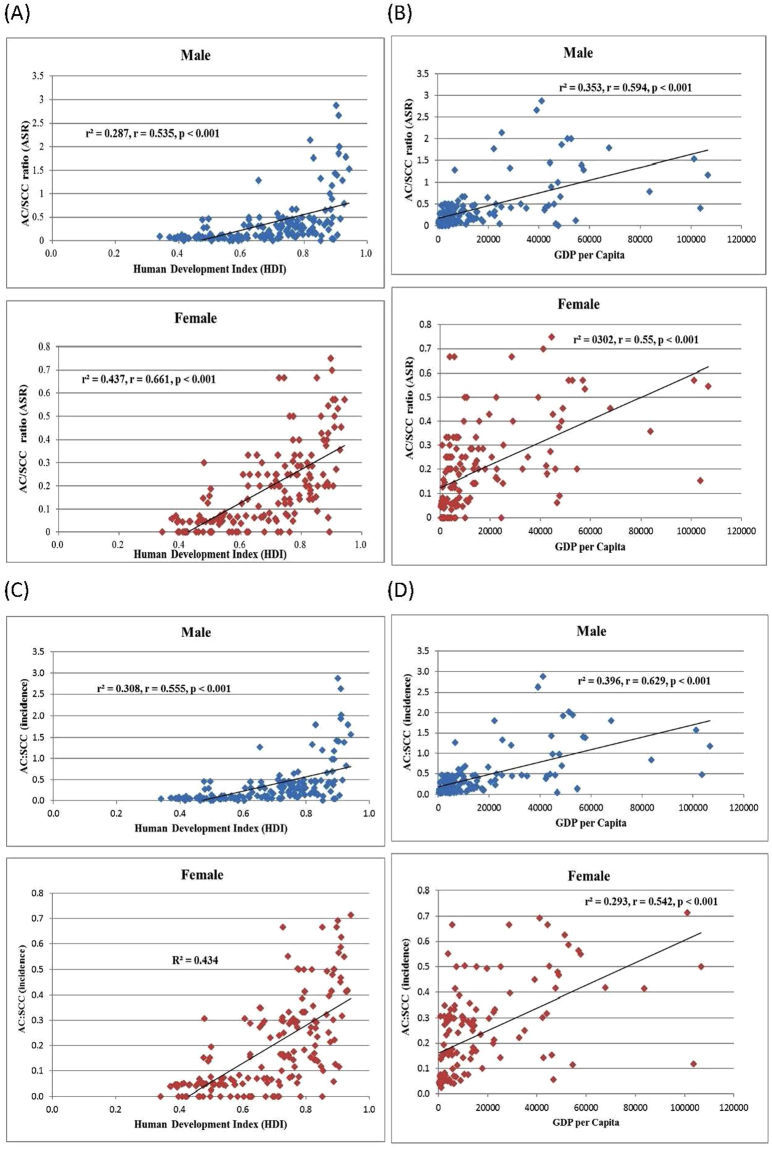
(**A**) Relationship between the ratio of age-standardised incidence rates of adenocarcinoma (AC): squamous cell carcinoma (SCC) and Human Development Index in male (upper panel) and female (lower panel). (**B**) Relationship between the ratio of age-standardised incidence rates of adenocarcinoma (AC): squamous cell carcinoma (SCC) and Gross Domestic Product per capita in male (upper panel) and female (lower panel). (**C**) Relationship between the ratio of crude incidence rates of adenocarcinoma (AC): squamous cell carcinoma (SCC) and Human Development Index in male (upper panel) and female (lower panel) (**D**) Relationship between the ratio of crude incidence rates of adenocarcinoma (AC): squamous cell carcinoma (SCC) and Gross Domestic Product per capita in male (upper panel) and female (lower panel).

The incidence of AC increased with HDI in men (r^2^ = 0·224, r = 0·473) and women (r^2^ = 0·037, r = 0·192), and similarly for its relationship with GDP (r^2^ = 0·288, r = 0·537 and r^2^ = 0·0716, r = 0·268 for male and female, respectively). For SCC, its incidence was inversely correlated with HDI (r^2^ = 0·051, r = −0·226, p = 0·004 for men; r^2^ = 0·098, r = −0·313, p < 0·001 for women) and GDP per capita (r^2^ = 0·040, r = −0·200, p = 0·012 for men; r^2^ = 0·0437, r = −0·209, p = 0·009 for women) (Fig. 1A to D). HDI was correlated with the ratio between the standardized incidence of AC and SCC (r² = 0·287, r = 0·535 [men]; r² = 0·437, r = 0·661 [women]) and GDP per capita (r² = 0·353, r = 0·594 [men]; r² = 0·302, r = 0·550 [women], all p < 0·001). Similar trend was observed when the ratio between the absolute incidence of AC and SCC was used (Fig. 2A to D).

### Time trends

The global trends of incidence and mortality according to gender were shown in Supplementary Figure 1. Among male subjects, 3 countries reported increasing incidence, 7 countries reported decreasing incidence, and 31 countries had stable rates of incidence. Among women, one country showed increasing incidence and 4 showed declining incidence. Turning to mortality rates, 2 countries had increasing trends, 10 countries reported decreasing male mortality and 29 nations reported relatively stable time trends in male subjects. Five countries had decreasing death rates and 36 countries had stable death rates in female populations.

#### Latin America/the Caribbean

**Figure 3:**
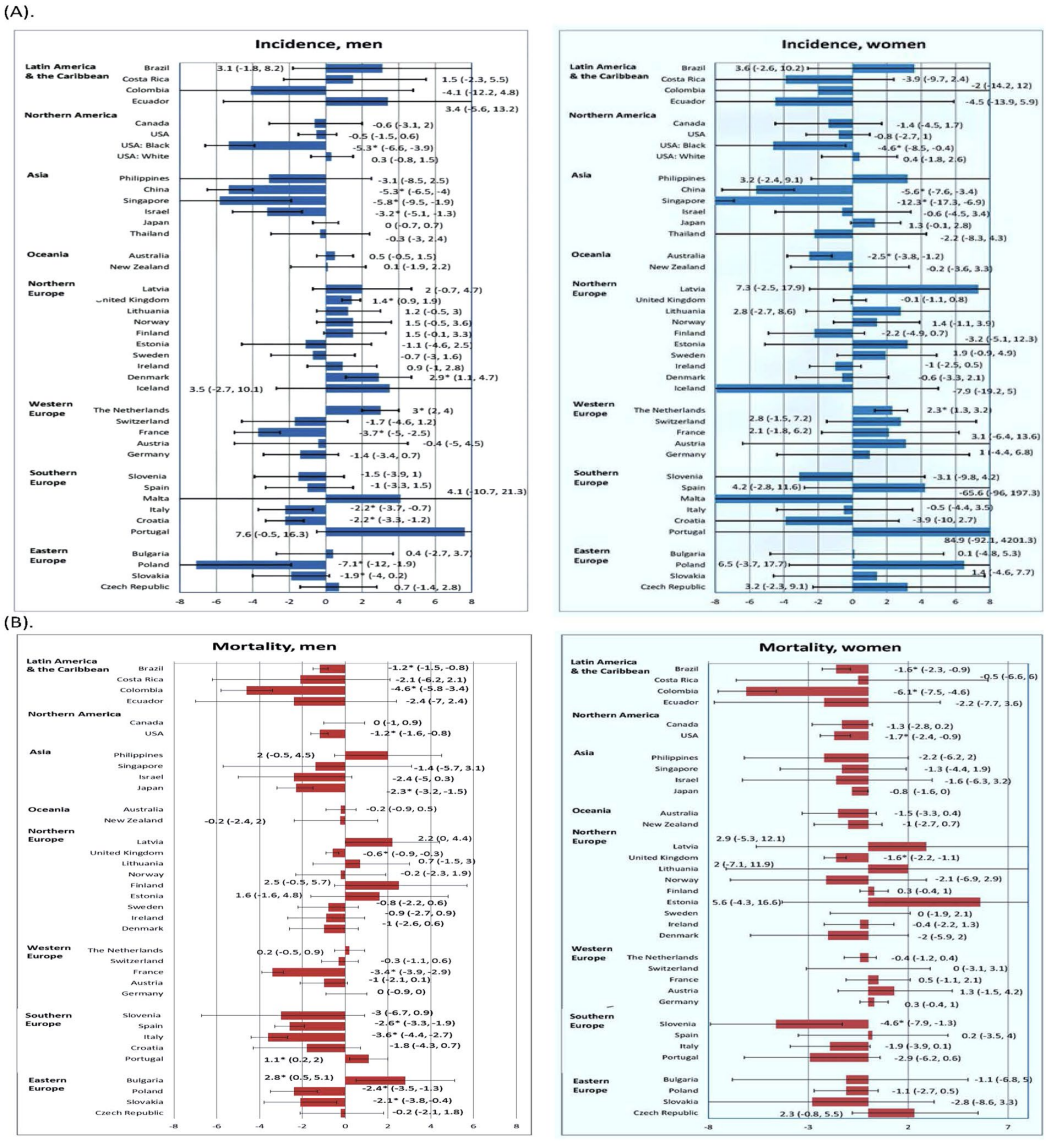
(**A**) Incidence trend of oesophageal cancer in male (left panel) and female (right panel). (**B**) Mortality trend of oesophageal cancer in male (left panel) and female (right panel).

All countries in this continent had stable incidence trends in the past decade of analysis (Fig. 3A). The ASR mortality rate reported a decline in Brazilian men (AAPC −1.2, 95% C.I. −1.5, −0.8) and women (AAPC −1.6, 95% C.I. −2.3, −0.9). Columbia also had a significant reduction in mortality among men (AAPC −4.6, 95% C.I. −5.8, −3.4) and women (AAPC −6.1, 95% C.I. −7.5, −4.6) (Fig. 3B).

#### Northern America

A reduction in incidence was reported in male (AAPC −5·3, 95% C.I. −6·6, −3·9) and female (AAPC −4·6, 95% C.I. −8·5, −0·4) Black American individuals. A modest reduction in death rates was observed among male populations (AAPC −1·2, 95% C.I. −1·6, −0·8) and women (AAPC −1·7, 95% C.I. −2·4, 0·9) in the United States irrespective of ethnicity.

#### Asia

China (AAPC −5·3, 95% C.I. −6·5, −4), Singapore (AAPC −5·8, 95% C.I. −9·5, −1·9) and Israel (AAPC −3·2, 95% C.I. −5·1, −1·3) reported an incidence reduction in male, and the former two also experienced a decrease in female (China: AAPC −5·6, 95% C.I. −7·6, −3·4; Singapore: AAPC −12·3, 95% C.I. −17·3, −6·9). There was a substantial decline in mortality in Japanese men (AAPC −2·3, 95% C.I. −3. 2, −1·5)  and women (AAPC −0.8, 95% C.I. −1.6, 0).

#### Oceania

The incidence decreased in Australia in female subjects (AAPC −2·5, 95% C.I. −3·8, −1·2). All other trends were stable.

#### Northern Europe

Only the United Kingdom (AAPC 1·4, 95% C.I.0·9, 1·9) and Denmark (AAPC 2.9, 95% C.I. 1.1, 4.7) reported an incidence increase among male. The United Kingdom also showed a mortality reduction among both male (AAPC −0·6, 95% C.I. −0·9, −0·3) and female (AAPC −1·6, 95% C.I. −2·2, −1·1). The majority of the countries had stable incidence and mortality rates.

#### Western Europe

The incidence rate was found to increase in the Netherlands in male populations (AAPC 3, 95% C.I. 2, 4) and female (AAPC 2·3, 95% C.I. 1·3, 3·2) individuals. France reported a decline in incidence (AAPC −3·7, 95% C.I. −5, −2·5) and mortality rates (AAPC −3·4, 95% C.I. −3·9, −2·9) among men.

#### Southern Europe

Italy and Croatia were two countries that had declines in incidence and mortality rates in male populations. Slovenia showed a decline in mortality among women (AAPC −4·6, 95% C.I. −7·9, −1·3).

#### Eastern Europe

Poland (AAPC −7·1, −12, 1·9) reported a substantial reduction in incidence in men.  Among men, Bulgaria (AAPC 2.8, 95% C.I. 0.5, 5.1) had a rise in mortality while Poland (AAPC −2.4, 95% C.I. −3.5, −1.3) and Slovakia (AAPC −2.1, 95% C.I. −3.8, −0.4) reported a decline in mortality.

## Discussion

This study evaluated the worldwide incidence/mortality rates of oesophageal cancer based on existing data. It was found that the incidence and mortality showed wide variations on a global basis. In men, Eastern Asia, Southern Africa and Eastern Africa had the highest number of new diagnoses - mostly SCC. The greatest mortality in male was found in Middle Africa, Western Africa and Eastern Asia. The highest incidence: mortality ratio was reported in Northern and Southern Africa among male, while in Western Europe and Southern America among female.

We found that HDI and GDP per capita were both correlated with AC:SCC ratios in incidence. The correlation coefficients for HDI and GPD *per capita* for the incidence of oesophageal cancer are large. One of the important findings includes the incidence decline in Black America, Singapore and China in men and women. In men, mortality rates increased drastically in less developed nations including the Philippines and Bulgaria. In women, a substantial decrease in mortality was reported for Columbia and Slovenia.

Findings from the present study corroborate those of previous literature. Populations in Asia such as Northern Iran^28^, Central Asia and China^29^, as well as Eastern Africa^30–32^, have high incidence rates - so that these regions were collectively named as the “oesophageal cancer belt”^10^. The higher incidence rates in these regions may reflect the changing epidemiology of the two histologic types of esophageal cancer (SCC vs. AC) in recent years, such as in Iran where the rates of AC were among the highest in the Central Asian region, particularly in women^33^. Some nations reported greater rates of AC when compared with that of SCC, including Northern/Western Europe and Northern America.

This study showed that nations with higher socioeconomic developement had higher AC:SCC ratio in correlation analyses. Poor oral hygiene and poverty have been associated with a higher SCC risk. In addition, there has been rising prevalence of obesity (a risk factor for AC)^10^ but declining tobacco smoking (risk of SCC) in more developed nations, which may underpin these correlation trends. Probable explanations for the reduction of mortality trends may consist of technological advancement of oesophageal cancer, such as improvements in endoscopic detection and therapy for early stage disease. These consist of minimally invasive esophagectomy^3^–which when accompanied by a strategy with conjoint use of laparoscopic and thoracoscopic techniques, can lead to a very low rate of postoperative mortality (1.7% within 30 days) and a reduction in pulmonary complications, when compared with open surgery. With the advances in endoscopic surveillance programs after Barrett’s esophagus is diagnosed, physicians could detect more early stage cancer and this could facilitate earlier management^3^. However, some countries including Bulgaria and the Philippines showed escalating rates of cancer death in women. This observation could be explained by the capacities and accessibility of healthcare services which are crucial for early diagnosis and management. The exact reasons on the rise of mortality rates in these countries are yet to be explored. It should be noted that the AC:SCC ratio only exceeded 1 in men in most developed nations. SCC was still a predominate type of oesophageal cancer as reported in this study. Arnold and colleagues^10^ previously analyzed the total numbers of AC and SCC in 2012, and reported that SCC was a disproportionally common histologic type when compared to AC on a global basis.

This study updated the incidence and mortality of oesophageal cancer, and explored its time trends and global variability by high quality data from recognized databases. Nonetheless, this study has some limitations. Firstly, cancer diagnoses could be under-reported and may induce bias in cancer registration, particularly in nations where resources are limited^29^. On the other hand, in nations where figures were derived from only one cancer registry situated in more developed areas, the estimates might have overestimated the real figures especially in those countries with large areas of underdeveloped regions. Also, less than half of the world’s nations provided high quality incidence/mortality data. Therefore, the incidence and mortality figures could have a certain degree of bias, especially in underprivileged nations.

In summary, the incidence of esophageal cancer decreased in the majority of nations, yet the death rates in male populations increased in some countries, especially in the Portugal and Bulgaria. With global expansion in population size, a further increase in its global health burden could be huge – particularly adenocarcinoma that is positively correlated with socioeconomic development. Therefore, more expenditure will be required to sustain its diagnosis and treatment. In the community, more resources should also be committed to primary prevention strategies.

## Electronic supplementary material

Supplementary Information 

## References

[1] Ferlay, J., Soerjomataram, I. & Ervik, M. *et al*. GLOBOCAN 2012v1.0, Cancer Incidence and Mortality Worldwide. IARC Cancer Base No. 11. Lyon, France: International Agency for Research on Cancer (2013).

[2] Di Pardol BJ (2016). The Global Burden of Esophageal Cancer: A Disability-Adjusted Life-Year Approach. World J Surg..

[3] Pennathur A, Gibson MK, Jobe BA, Luketich JD (2013). Oesophageal carcinoma. Lancet..

[4] Mao WM, Zheng WH, Long ZQ (2011). Epidemiologic risk factors for esophageal cancer development. Asian Pac J Cancer Prev..

[5] Lepage C, Rachet B, Jooste V, Faivre J, Coleman MP (2008). Continuing rapid increase in esophageal adenocarcinoma in England and Wales. Am J Gastroenterol..

[6] Eslick GD (2009). Epidemiology of esophageal cancer. Gastroenterol Clin N Am..

[7] Zhang Y (2013). Epidemiology of esophageal cancer. World J Gastroenterol..

[8] Edgren G, Adami HO, Weiderpass E, Nyren O (2013). A global assessment of the oesophageal adenocarcinoma epidemic. Gut..

[9] Holmes RS, Vaughan TL (2006). Epidemiology and pathogenesis of esophageal cancer. Semin Radiat Oncol..

[10] Arnold M, Soerjomataram I, Ferlay J, Forman D (2015). Global incidence of oesophageal cancer by histologic subtype in 2012. Gut..

[11] Wong, M. C. *et al*. The global epidemiology of bladder cancer: a joinpoint regression analysis of its incidence and mortality trends and projection. *Sci Rep* (in press, 2018) 10.1038/s41598-018-19199-z PMC5773684 29348548

[12] Wong MC (2016). Global incidence and mortality of prostate cancer: analysis of temporal patterns and trends in 36 countries. Eur Urol..

[13] Wong MC (2017). Global temporal patterns of pancreatic cancer and association with socioeconomic development. Sci Rep.

[14] Wong MC (2017). International incidence and mortality trends of liver cancer: a global profile. Sci Rep.

[15] Human Development Report 2013. The rise of the south: human progress in a diverse world. New York: United Nations Development Programme (UNDP) (2013).

[16] Forman, D. *et al*. Cancer Incidence in Five Continents, Vol. X (electronic version). Lyon: IARC, http://ci5.iarc.fr(2013).

[17] SEER. SEER*Stat Database: Incidence—SEER 9 Regs Research Data, November 2013 Sub (1992–2011)Surveillance, Epidemiology, and End Results (SEER) rogram. http://www.seer.cancer.gov (2013).

[18] Steliarova-Foucher, E. *et al*. European Cancer Observatory: Cancer Incidence, Mortality, Prevalence and Survival in Europe. Version 1.0. European Network of Cancer Registries, International Agency for Research on Cancer. http://eco.iarc.fr (2012).

[19] Engholm, G. *et al*. NORDCAN: Cancer Incidence, Mortality, Prevalence and Survival in the Nordic Countries, Version 7.1. Association of the Nordic Cancer Registries. Danish Cancer Society. http://www.ancr.nu (2015).

[20] Quality Improvement Process. National Cancer Institute, Surveillance, Epidemiology, and End Results (SEER) Program. Available at: https://seer.cancer.gov/qi/process.html. Accessed on 12 September (2017).

[21] Steliarova-Foucher E (2015). The European Cancer Observatory: A new data resource. Eur J Cancer.

[22] Engholm G (2010). NORDCAN – a Nordic tool for cancer information, planning, quality control and research. Acta Oncologica.

[23] World Health Organization. International Classification of Diseases forOncology. 3r^d^ edn, First Revision. Geneva, Switzerland: World Health Organization (2013).

[24] Mathers CD (2005). Counting the dead and what they died from: an assessment of the global status of cause of death data. Bull World Health Organ..

[25] Segi M, Fujisaku S, Kurihara M (1957). Geographical observation on cancer mortality by selected sites on the basis of standardised death rate. Gan..

[26] Kim HJ (2000). Permutation tests for joinpoint regression with applications to cancer rates. Stat Med..

[27] Clegg LX (2009). Estimating average annual percent change in trend analysis. Stat Med..

[28] Mosavi-Jarrahi A, Mohagheghi MA (2006). Epidemiology of esophageal cancer in the high-risk population of Iran. Asia Pac J Cancer Prev..

[29] Lin Y (2013). Epidemiology of esophageal cancer in Japan and China. J Epidemiol..

[30] Ocama P (2008). Factors associated with carcinoma of the oesophagus at Mulago Hospital, Uganda. Afr Health Sci..

[31] Somdyala NI (2010). Cancer incidence in a rural population of South Africa, 1998-2002. Int J Cancer..

[32] Vizcaino AP, Parkin DM, Skinner ME (1995). Risk factors associated with oesophageal cancer in Bulawayo, Zimbabwe. Br J Cancer..

[33] Ghasemi-Kebria F (2013). Marked increase in the incidence rate of esophageal adenocarcinoma in a high-risk area for esophageal cancer. Arch Iran Med..

