# Supplementary material for: Global Incidence and mortality of oesophageal cancer and their correlation with socioeconomic indicators temporal patterns and trends in 41 countries
Source: Sci Rep. 2018 Mar 14;8:4522. doi: 10.1038/s41598-018-19819-8 (PMC5852053; doi:10.1038/s41598-018-19819-8)
Supplement: Supplementary file 1 — Supplementary Information [file 41598_2018_19819_MOESM1_ESM.pdf]

Global Incidence and mortality of oesophageal cancer and their correlation with socioeconomic indicators: temporal patterns and trends in 41 countries

Martin CS Wong MD[a, b, i], Willie Hamilton PhD[c], David C Whiteman PhD[d], Johnny Y Jiang PhD[e], Youlin Qiao PhD[e], Franklin DH Fung BHSc (Hons)[a], Harry HX Wang PhD[f, g], Philip WY Chiu MD[b], Enders KW Ng MD[b], Justin CY Wu MD[b, h, i], Jun Yu PhD[b, h, i], Francis KL Chan MD[b, h, i], Joseph JY Sung MD[b, h, i]

a. School of Public Health and Primary Care, Faculty of Medicine, the Chinese University of Hong Kong.

b. Institute of Digestive Disease, Faculty of Medicine, the Chinese University of Hong Kong

c. University of Exeter, College House, St Luke's Campus, Exeter, United Kingdom;

d. QIMR Berghofer Medical Research Institute, Brisbane, Australia;

e. Peking Union School of Public Health, Chinese Academy of Medical Sciences and Peking Union Medical College, China

f. School of Public Health, Sun Yat-Sen University, Guangzhou, 510080, P.R. China;

g. General Practice and Primary Care, Institute of Health and Wellbeing, University of Glasgow

h. Department of Medicine and Therapeutics, Faculty of Medicine, Chinese University of Hong Kong

i. State Key Laboratory of Digestive Disease, Faculty of Medicine, Chinese University of Hong Kong

Correspondence during submission: Professor Martin CS Wong MD, MPH

Corresponding Author:

Professor Joseph JY Sung MD, PhD

Founding Director, Institute of Digestive Disease, Faculty of Medicine, Chinese University of Hong Kong;

7/F, Lui Che Woo Clinical Science Building, Prince of Wales Hospital, Shatin, NT, HKSAR.

Tel: 852 3943 8600; **Fax**: 852 2603 7301; **E-mail**: jjysung@cuhk.edu.hk

Supplementary Table 1 Data source for the age-standardized incidence and mortality rates of oesophageal cancer

	Incidence	Mortality
Austria	CI5 (1998-2007)	WHO (2005-2014)
Croatia	CI5 (1998-2007)	WHO (2004-2013)
Czech Republic	CI5 (1998-2007)	WHO (2004-2013)
Denmark	NORDCAN (2004-2013)	WHO (2003-2012)
Estonia	CI5 (2003-2012)	WHO (2003-2012)
Finland	NORDCAN (2004-2013)	WHO (2004-2013)
France	CI5 (1998-2007)	WHO (2002-2011)
Germany	CI5 (1998-2007)	WHO (2004-2013)
Iceland	NORDCAN (2004-2013)	N/A
Italy	CI5 (1998-2007)	WHO (1994-2003)
Latvia	CI5 (1998-2007)	WHO (2003-2012)
Lithuania	CI5 (1998-2007)	WHO (2004-2013)
Malta	CI5 (1998-2007)	N/A
Netherlands	CI5 (1998-2007)	WHO (2004-2013)
Norway	NORDCAN (2005-2014)	WHO (2004-2013)
Poland	CI5 (1997-2006)	WHO (2004-2013)
Slovakia	CI5 (1998-2007)	WHO (2001-2010)
Slovenia	CI5 (1998-2007)	WHO (2001-2010)
Spain	CI5 (1998-2007)	WHO (2004-2013)
Sweden	NORDCAN (2004-2013)	WHO (2004-2013)
Switzerland	CI5 (1998-2007)	WHO (2004-2013)
United Kingdom	CI5 (1998-2007)	WHO (2004-2013)
Australia	AIHW (2003-2012)	AIHW (2004-2013)
New Zealand	MHNZ (2003-2012)	MHNZ (2003-2012)
Bulgaria	EUREG (1998-2007)	WHO (2003-2012)
Ireland	EUREG (2000-2009)	WHO (2003-2012)
Brazil	CI5 (1998-2007)	WHO (2004-2013)
Colombia	CI5 (1998-2007)	WHO (2003-2012)
Costa Rica	CI5 (1998-2007)	WHO (2004-2013)
Ecuador	CI5(1998-2007)	WHO (2004-2013)
Canada	CI5(1998-2007)	WHO (2002-2011)
USA	NCI (1998-2007)	WHO (2004-2013)
USA White	NCI (1998-2007)	N/A
USA Black	NCI (1998-2007)	N/A

Israel	CI5 (1998-2007)	WHO (2004-2013)
Japan	CI5 (1998-2007)	WHO (2004-2013)
Philippines	CI5 (1998-2007)	WHO (1994-2003)
Singapore	CI5 (1998-2007)	WHO (2005-2014)
Thailand	CI5 (1998-2007)	N/A
China	CI5 (1998-2007)	N/A
Portugal	EUREG (2002-2011)	WHO (1994-2003)

'N/A' not available; AIHW: Australian Cancer Incidence and Mortality Books [1]; CI5: Cancer Incidence in Five Continents V [2]; EUREG: European Union Registration [3]; MHNZ: the Ministry of Health of New Zealand [4]; NORDCAN: Nordic Cancer Registries [5]; NCI: National Cancer Institution of the US [6]

Reference:

1. AIHW http://www.aihw.gov.au/acim-books/
2. CI5: http://ci5.iarc.fr/CI5plus/Pages/online.aspx
3. EUREG: http://eco.iarc.fr/eureg/AnalysisT.aspx
4. MHNZ: http://www.health.govt.nz/publication/cancer-historical-summary-1948-2012
5. NORDCAN: http://www-dep.iarc.fr/NORDCAN/english/frame.asp
6. NCI: http://seer.cancer.gov/faststats/selections.php?series=data

Supplementary Table 2 The methodology and data quality of data source

Data source	Methodology	Data quality
1. AIHW (Australian Cancer Incidence and Mortality Books)	• Reported national-level data from Australian Institute of Health and Welfare [1]	• Applied standard data quality measures [1]
2. CI5 (Cancer Incidence in five continents)	• Using population-based cancer registries throughout the world, obtaining incident rate by recording the occurrence of every case of cancer over a specified period [2]<br>• Covering around 21% of the world population in year 2006). [3]	• Used indices including the percentage of cases microscopically verified (MV%), the percentage of cases registered from a death certificate only (DCO%) and the ratio of the number of deaths to the number of cases registered (MI%) to examined data quality by volume, cancer site and sex [3]
3. EUREG (European Cancer Observatory)	• Reported data from national statistics bureaucracy (e.g. National Statistical Institute for Bulgaria; Census Enquiries and Demography Office for Ireland; National Office of Statistics for Portugal) [4]	• Applied standard data quality measures<br>• Over 11% of incident cancer were sent back to national registers for double-checks and corrections [5]
4. MHNZ (the Ministry of Health of New Zealand)	• Data were originated from national cancer registry, mortality collection, as well as the Cancer: New Registrations and Deaths and Mortality and Demographic data publications, and Medical Statistics in New Zealand [6]	• Applied international rules to ensure comparability

5. NORDCAN (Nordic Cancer Registries)	• Data were originated from the national cancer registries and causes of death registries according to international rules ensure comparability [7]	• Comprehensive and have been recognized as the standards for quality among cancer registries
6. WHO mortality database	• The cause-of-death statistics are from country civil registration systems. When a death occurs, the event is registered at the local civil registry with information on the cause of death. The information is then compiled by the national authority and submitted to WHO every year. Only medically-certified deaths were published by WHO. [8]<br><br>• All reported numbers are in age-specific rates, and were calculated into age-standard rates according to 1991 standard population. [8]	• Comprehensive and have been recognized as the standards for quality among cancer registries

[1] Australian Institute of Health and Welfare. Cancer in Australia: Actual incidence data from 1982 to 2013 and mortality data from 1982 to 2014 with projections to 2017. *Asia-Pacific Journal of Clinical Oncology* 14: 5-15(2017).

[2] Ferlay J, et al. Estimates of worldwide burden of cancer in 2008: GLOBOCAN 2008. *Int J Cancer* 127:2893-917 (2010).

[3] Parkin DM, et al. Fifty years of cancer incidence: CI5 I–IX. *Int J Cancer* 127:2918-27 (2010).

[4] Steliarova-Foucher, E. et al. European Cancer Observatory: Cancer Incidence, Mortality, Prevalence and Survival in Europe. Version 1.0. European Network of Cancer Registries, International Agency for Research on Cancer. http://eco.iarc.fr (2012).

[5] Blakely T, Shaw C. Cancer Trends: Trends in Cancer Incidence by Ethnic and Socioeconomic Group, New Zealand 1981-2004. University of Otago and the Ministry of Health; 2007.

[6] Steliarova-Foucher E, O'Callaghan M, Ferlay J, Masuyer E, Rosso S, Forman D, Bray F, Comber H. The European cancer observatory: a new data resource. *Eur J Cancer* 51:1131-43 (2015)

[7] Engholm G, Ferlay J, Christensen N, Bray F, Gjerstorff ML, Klint Å, Køtlum JE, Ólafsdóttir E, Pukkala E, Storm HH. NORDCAN–a Nordic tool for cancer information, planning, quality control and research. *Acta oncologica* 49:725-36 (2010)

[8] World Health Organization. International Classification of Diseases for Oncology. 3rd edn, First Revision. Geneva, Switzerland: World Health Organization (2013).

Supplementary Figure 1 Temporal trends in the incidence and mortality of esophageal cancer according to country

1). Latin America and the Caribbean

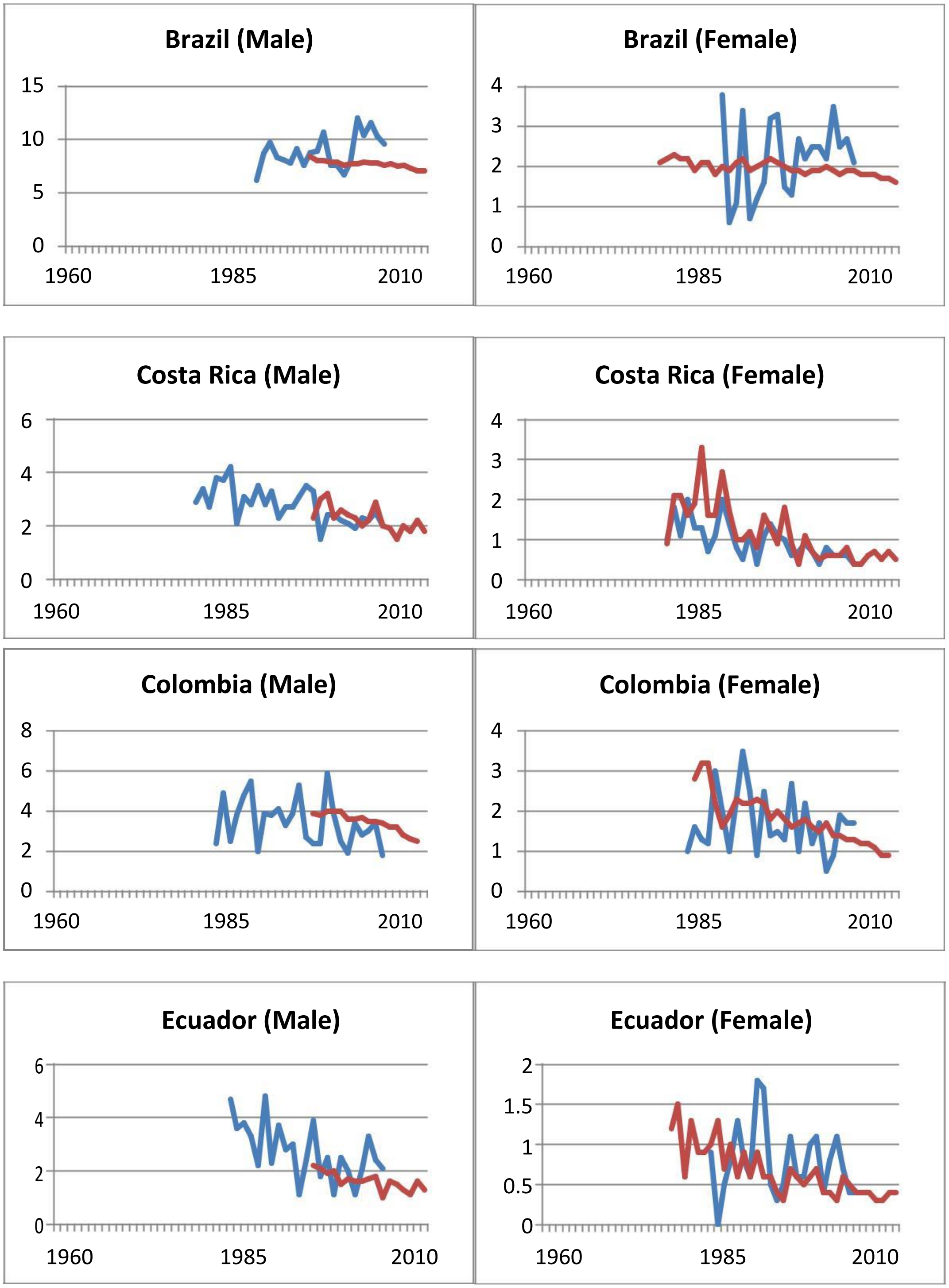

2). Northern America

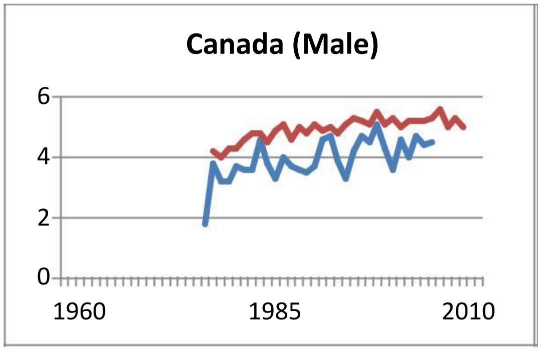

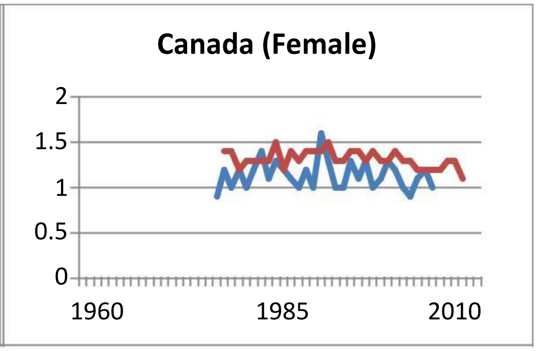

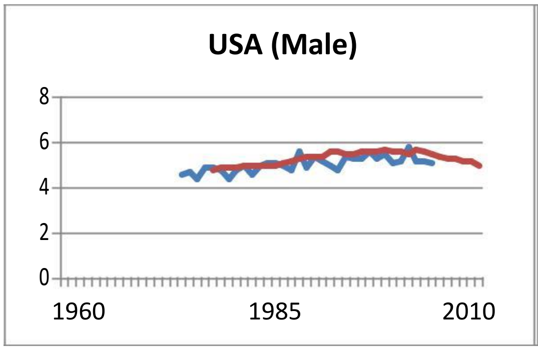

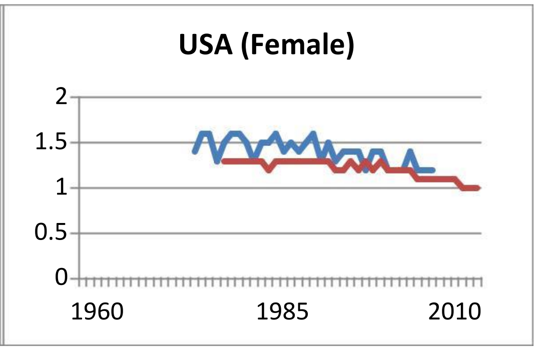

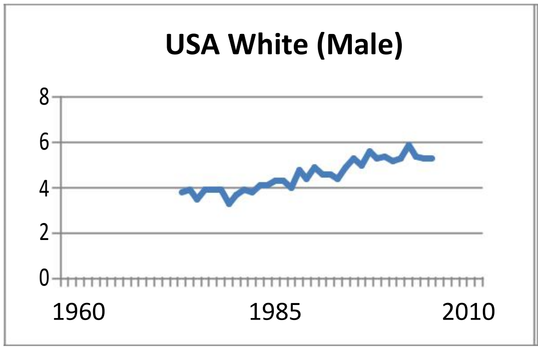

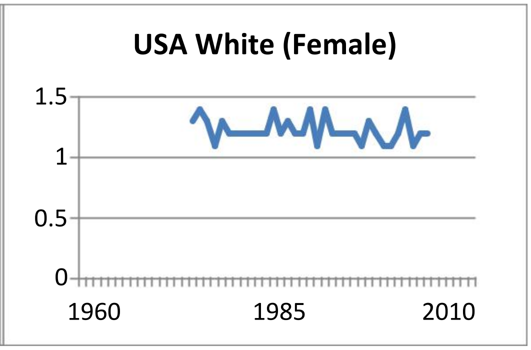

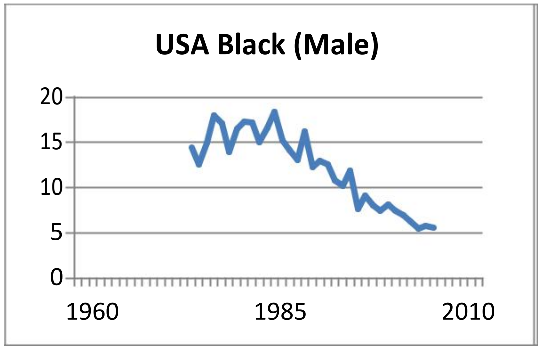

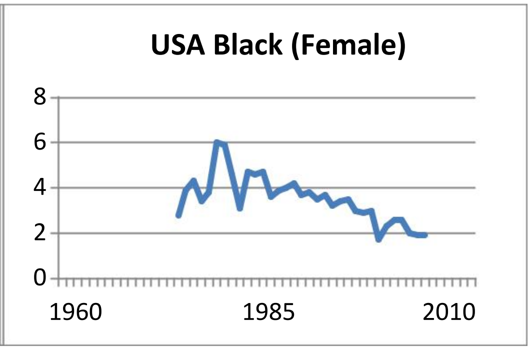

3). Asia

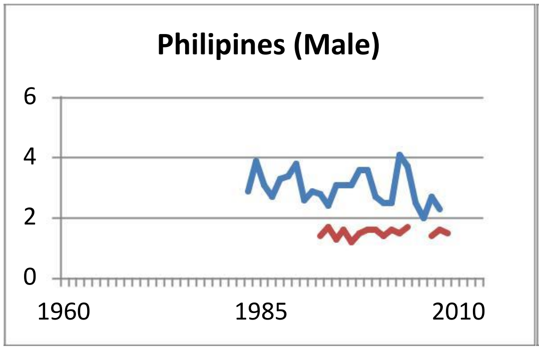
Philipines (Male)
6
4
2
0
1960
1985
2010

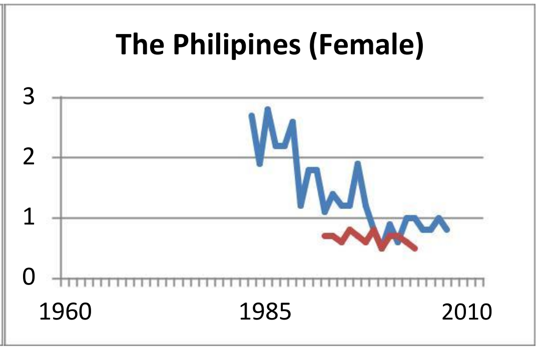
The Philipines (Female)
3
2
1
0
1960
1985
2010

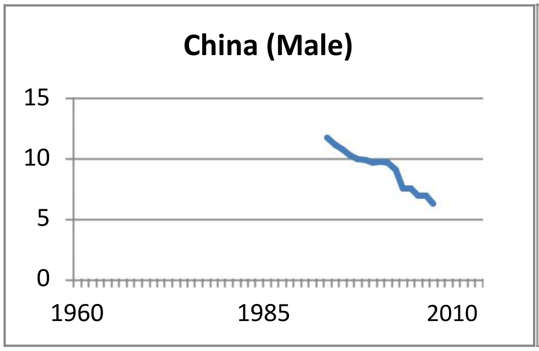
China (Male)
15
10
5
0
1960
1985
2010

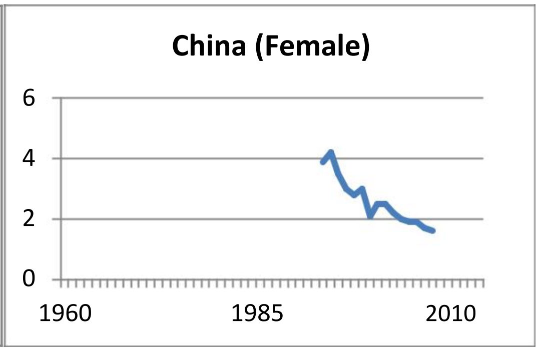
China (Female)
6
4
2
0
1960
1985
2010

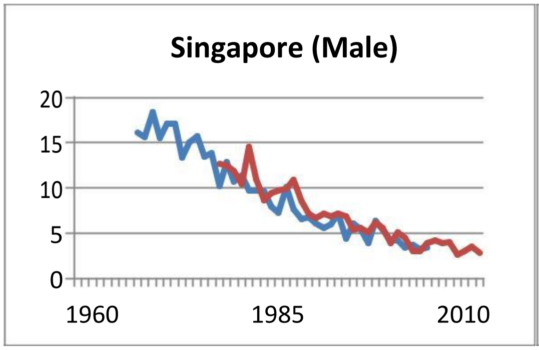
Singapore (Male)
20
15
10
5
0
1960
1985
2010

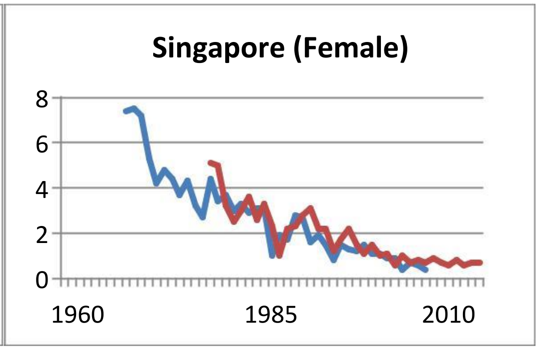
Singapore (Female)
8
6
4
2
0
1960
1985
2010

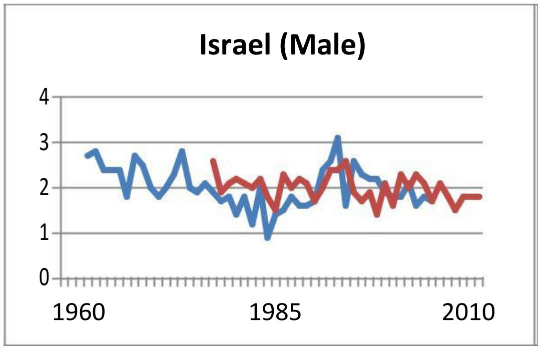
Israel (Male)
4
3
2
1
0
1960
1985
2010

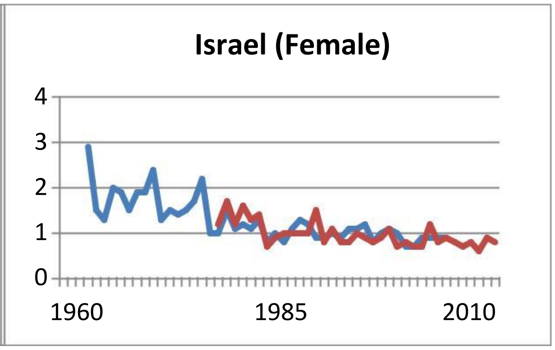
Israel (Female)
4
3
2
1
0
1960
1985
2010

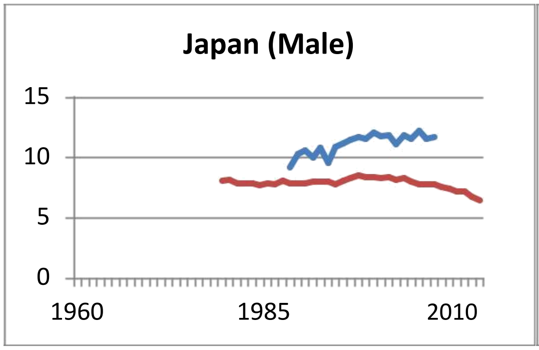
Japan (Male)
15
10
5
0
1960
1985
2010

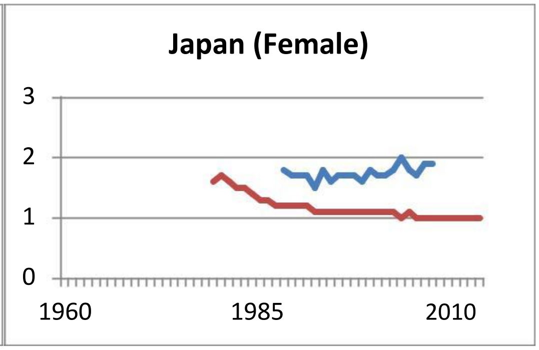
Japan (Female)
3
2
1
0
1960
1985
2010

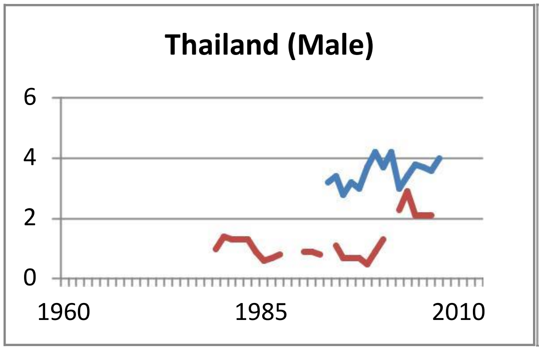
Thailand (Male)
6
4
2
0
1960
1985
2010

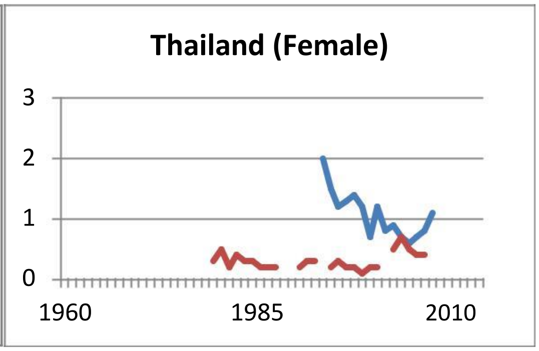
Thailand (Female)
3
2
1
0
1960
1985
2010

4). Oceania

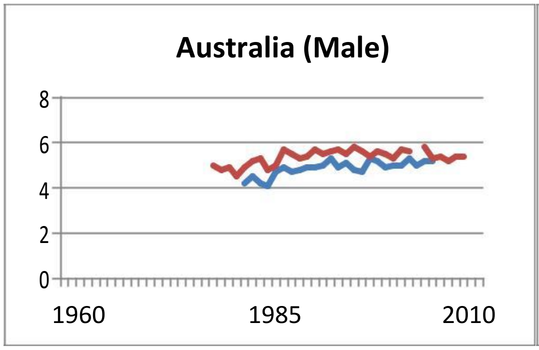
Australia (Male)
8
6
4
2
0
1960
1985
2010

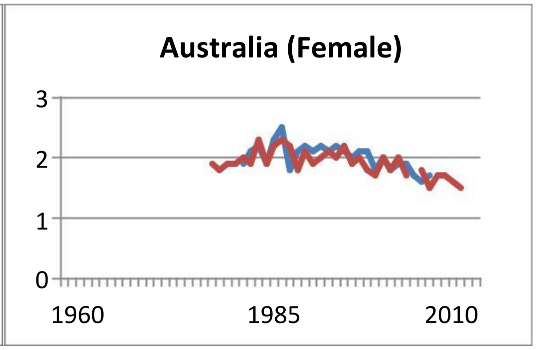
Australia (Female)
3
2
1
0
1960
1985
2010

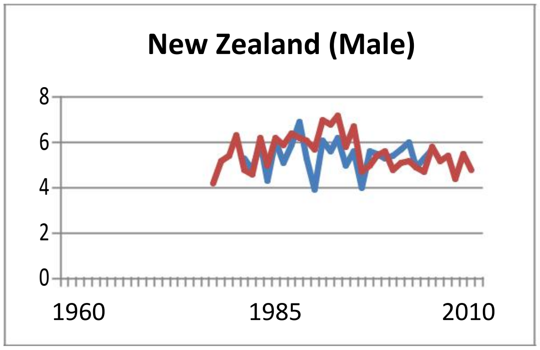
New Zealand (Male)
8
6
4
2
0
1960
1985
2010

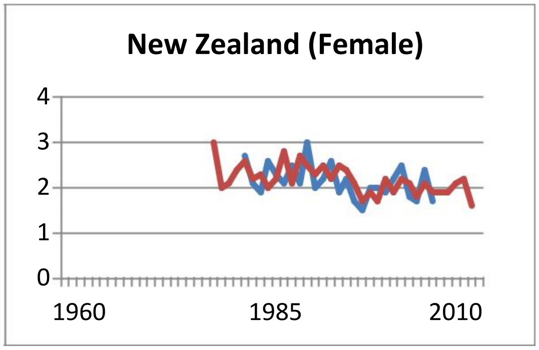
New Zealand (Female)
4
3
2
1
0
1960
1985
2010

5). Northern Europe

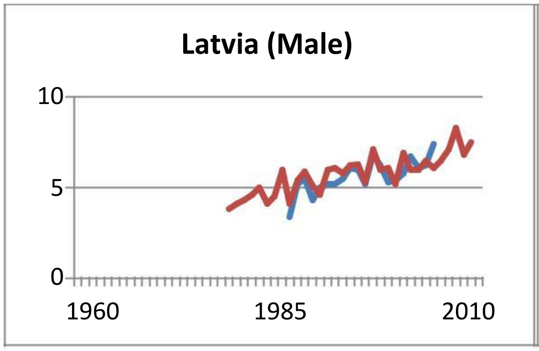

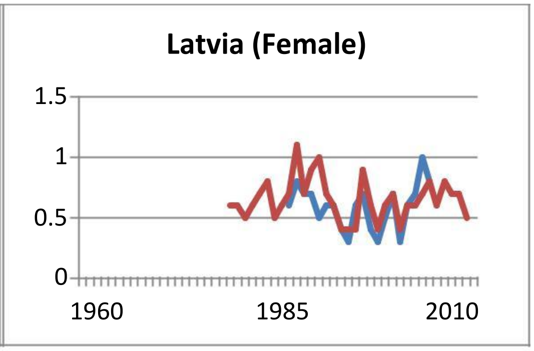

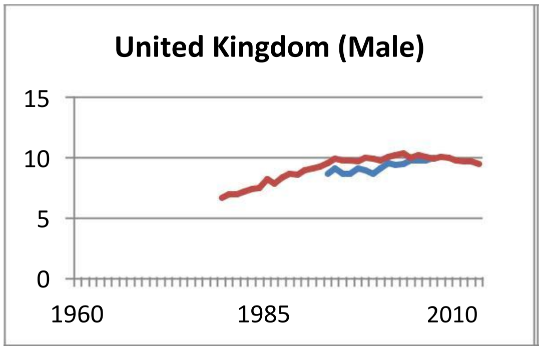

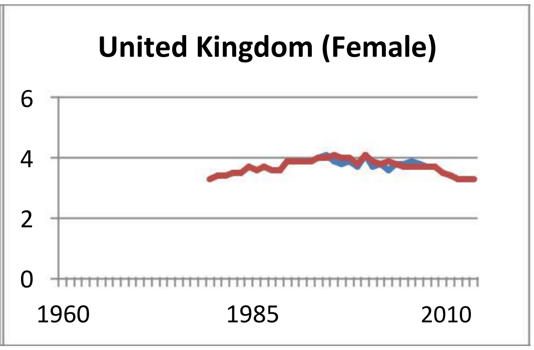

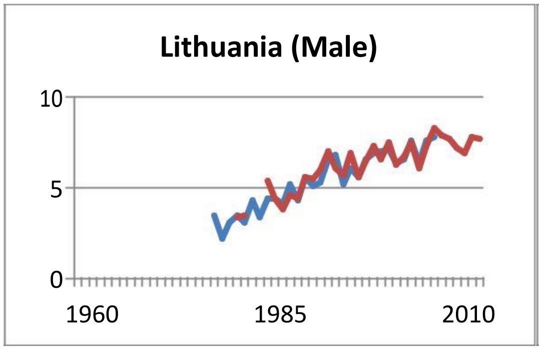

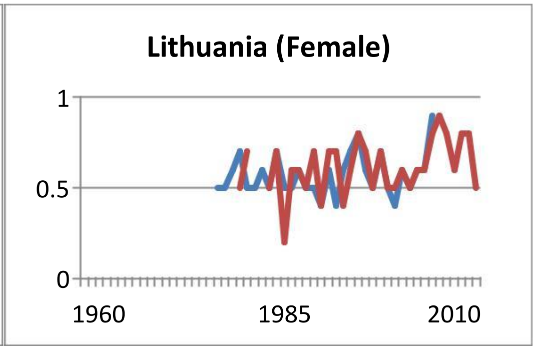

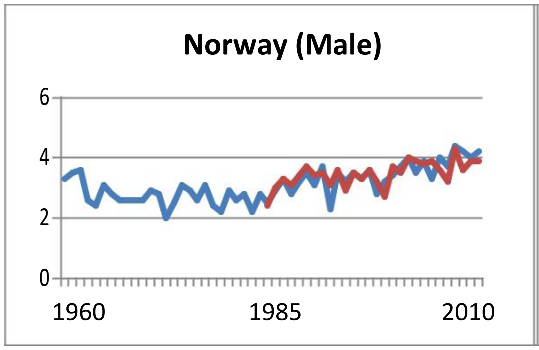

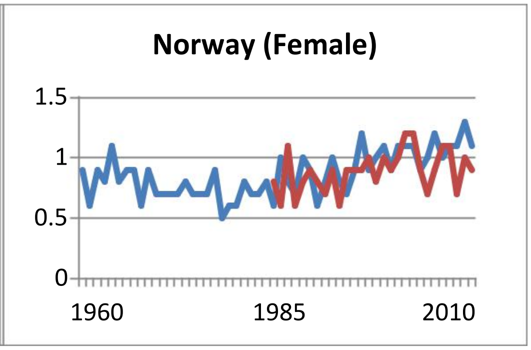

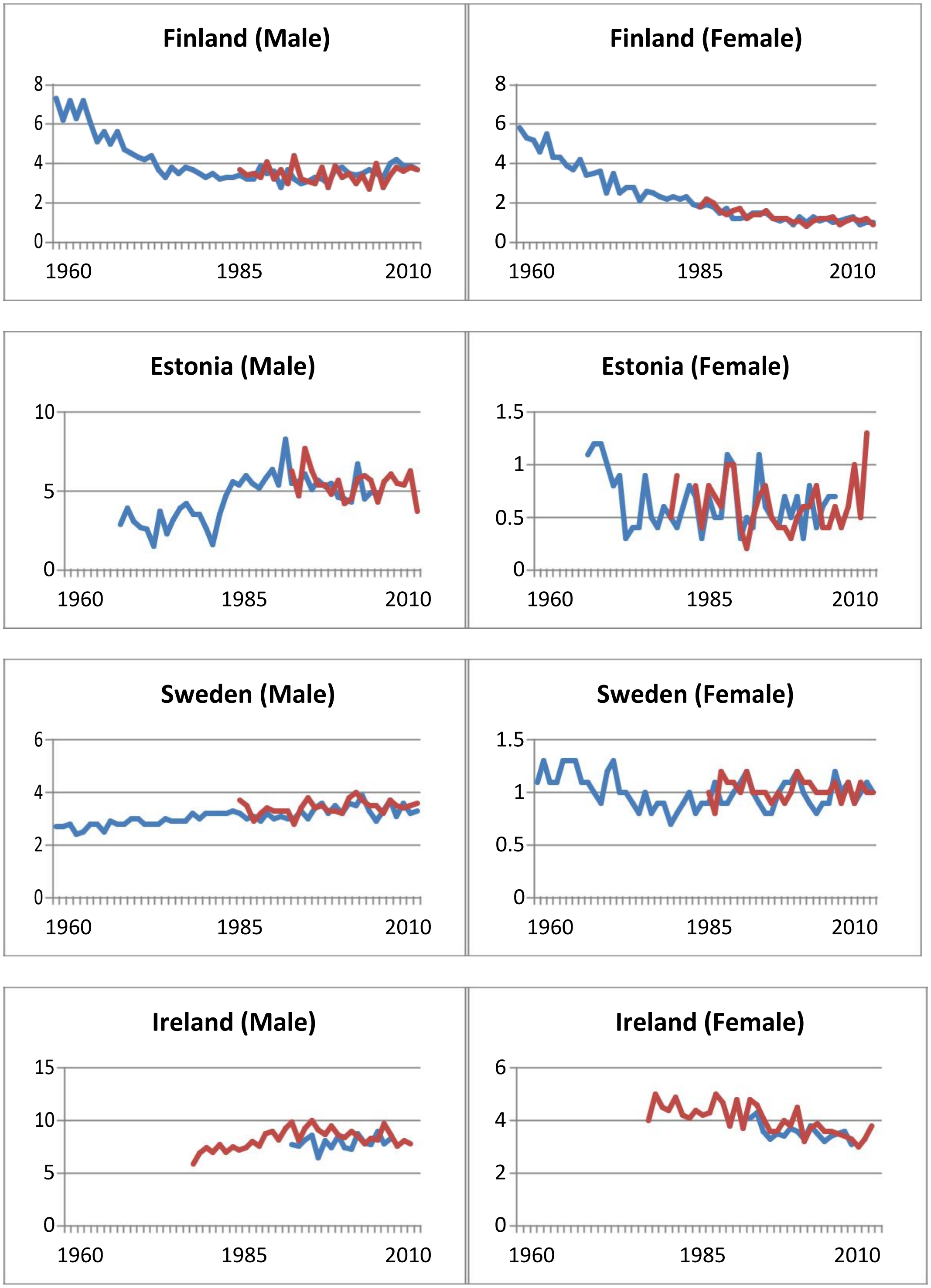

Finland (Male)
8
6
4
2
0
1960
1985
2010
Finland (Female)
8
6
4
2
0
1960
1985
2010
Estonia (Male)
10
5
0
1960
1985
2010
Estonia (Female)
1.5
1
0.5
0
1960
1985
2010
Sweden (Male)
6
4
2
0
1960
1985
2010
Sweden (Female)
1.5
1
0.5
0
1960
1985
2010
Ireland (Male)
15
10
5
0
1960
1985
2010
Ireland (Female)
6
4
2
0
1960
1985
2010

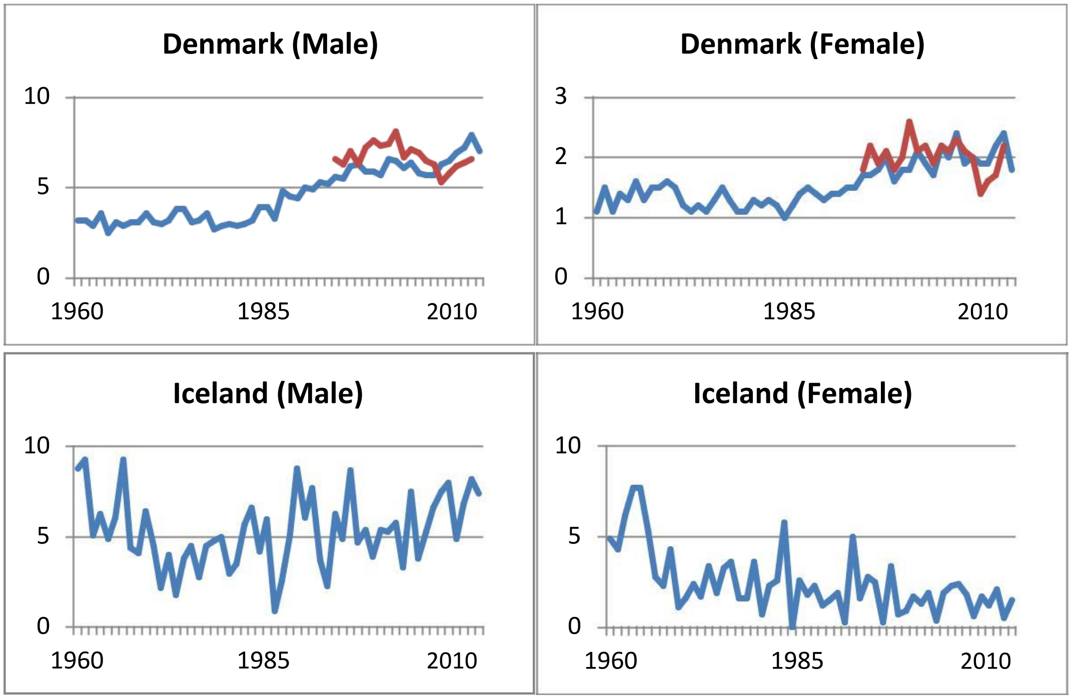

6). Western Europe

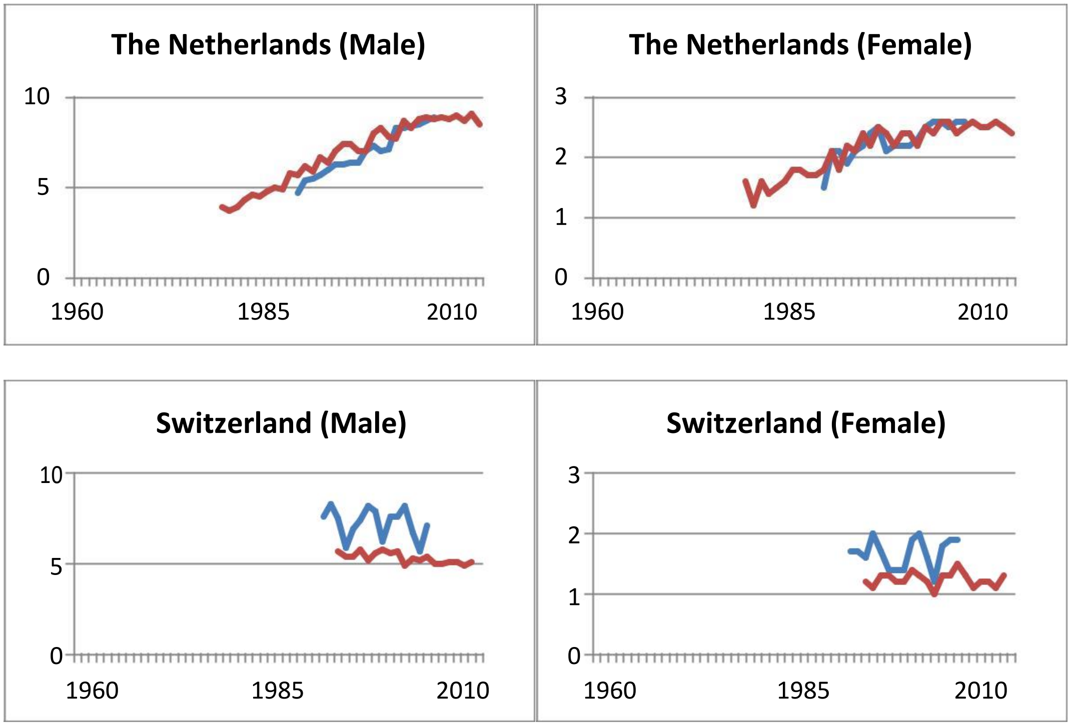

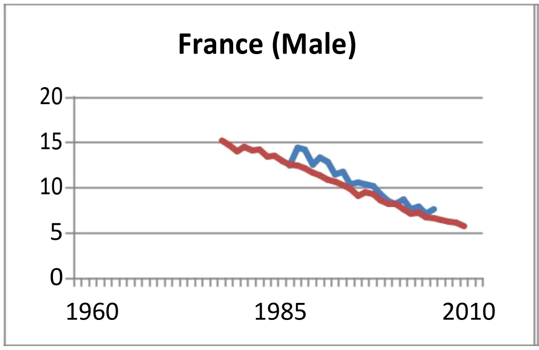

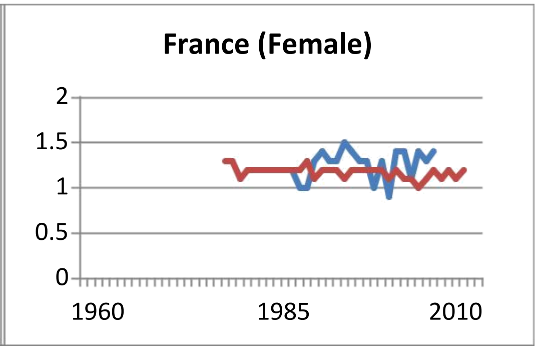

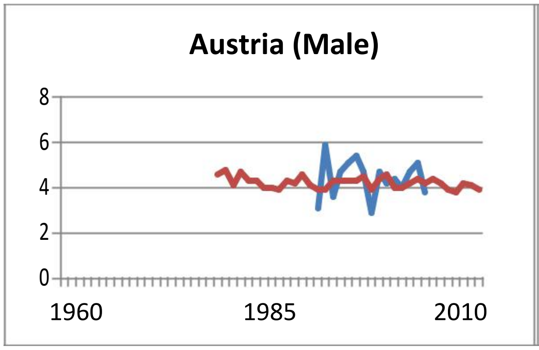

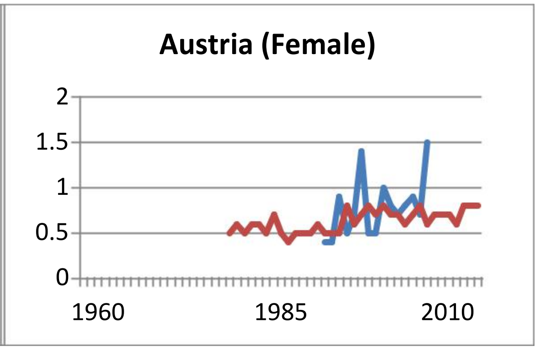

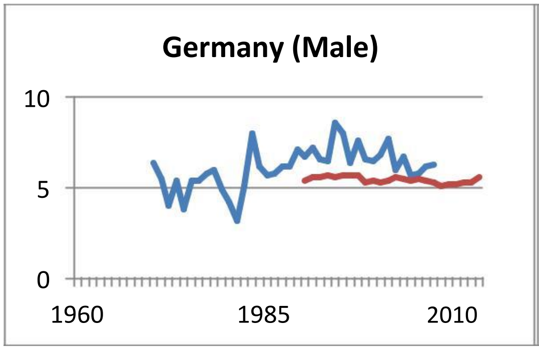

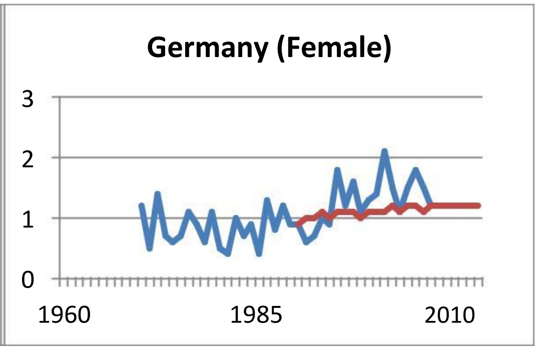

7). Southern Europe

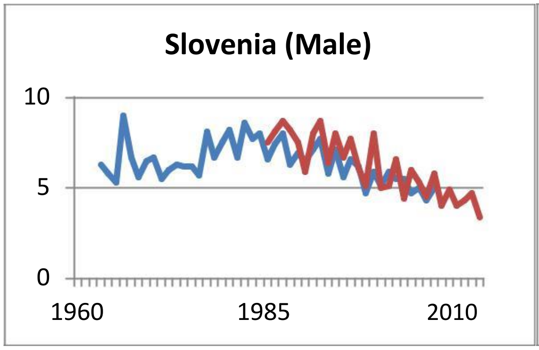

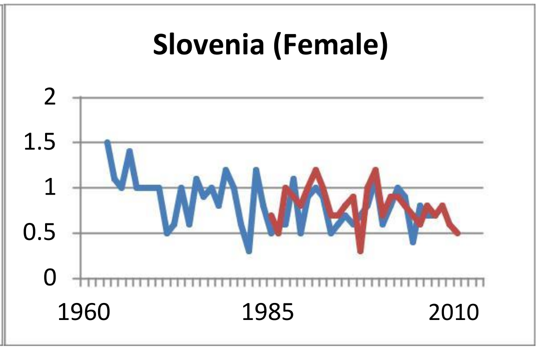

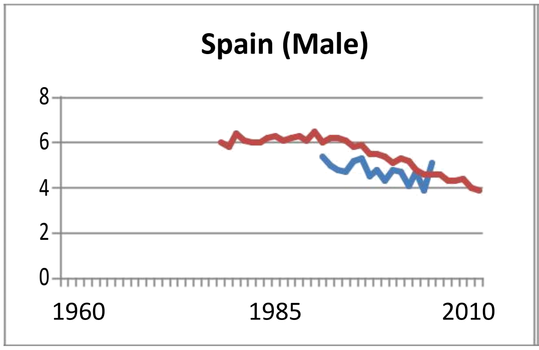

Spain (Male)
8
6
4
2
0
1960
1985
2010

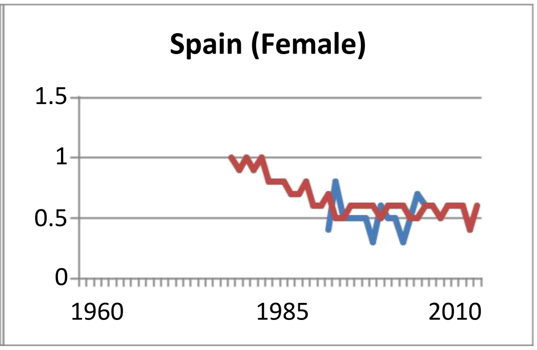

Spain (Female)
1.5
1
0.5
0
1960
1985
2010

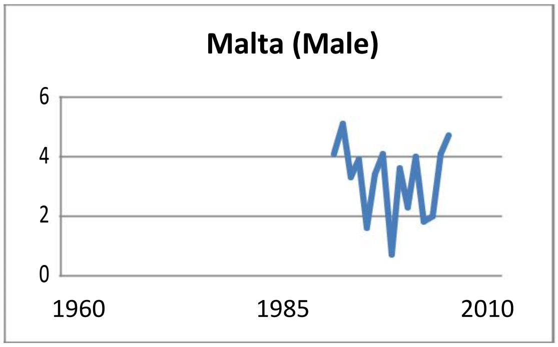

Malta (Male)
6
4
2
0
1960
1985
2010

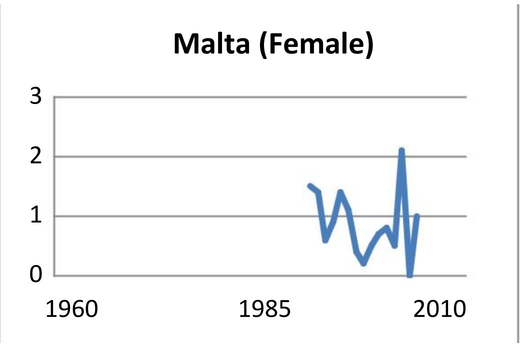

Malta (Female)
3
2
1
0
1960
1985
2010

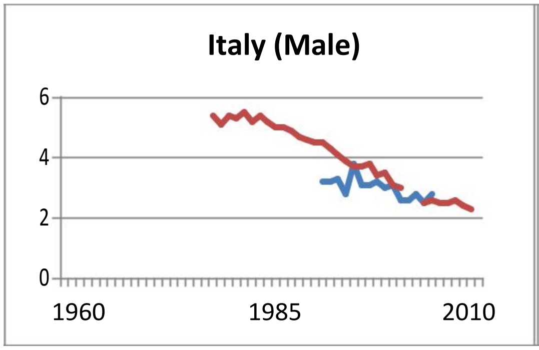

Italy (Male)
6
4
2
0
1960
1985
2010

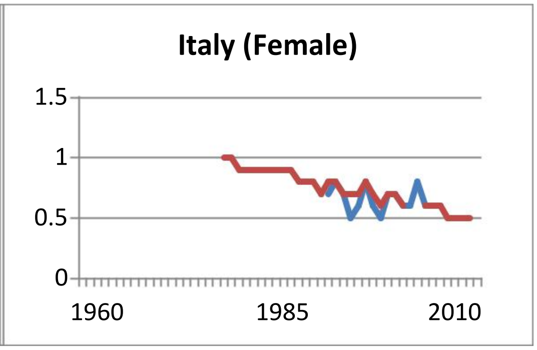

Italy (Female)
1.5
1
0.5
0
1960
1985
2010

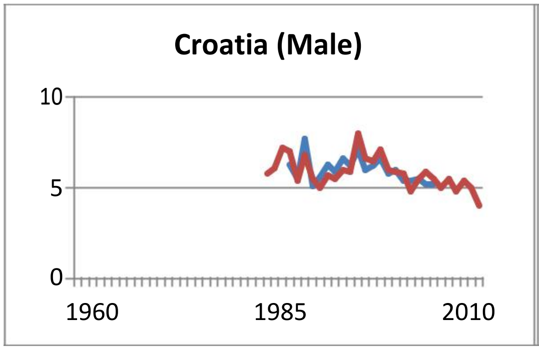

Croatia (Male)
10
5
0
1960
1985
2010

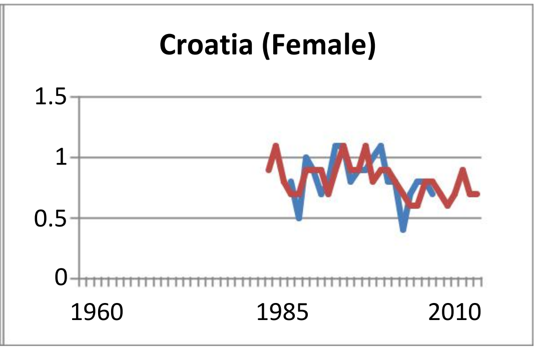

Croatia (Female)
1.5
1
0.5
0
1960
1985
2010

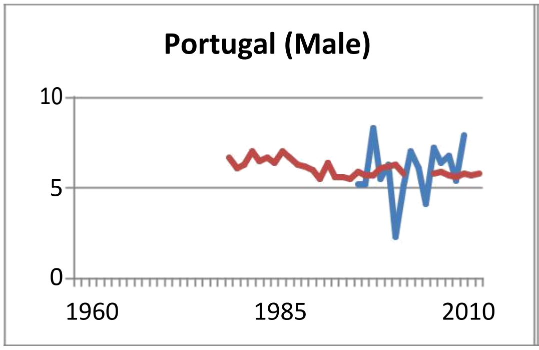

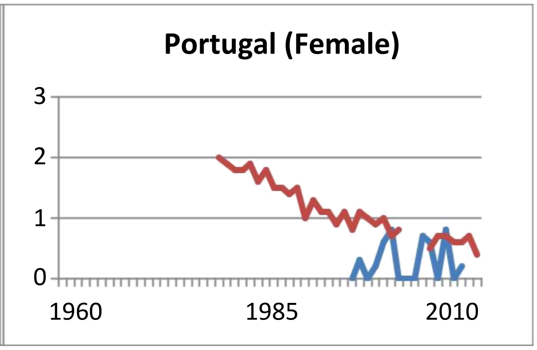

8). Eastern Europe

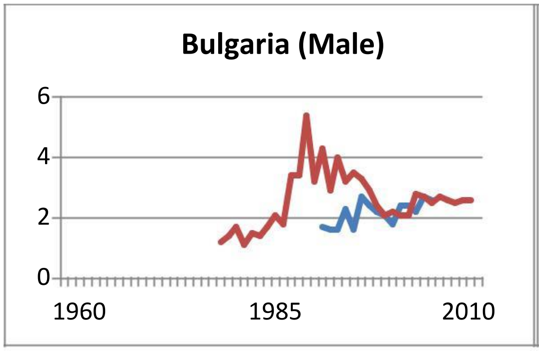

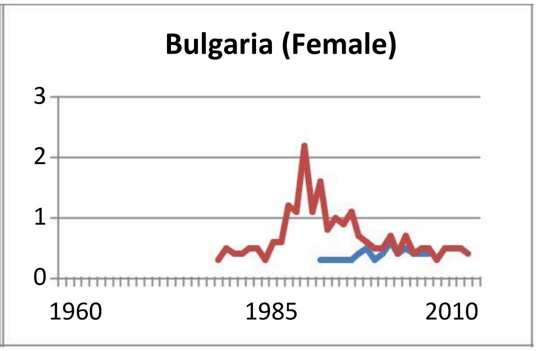

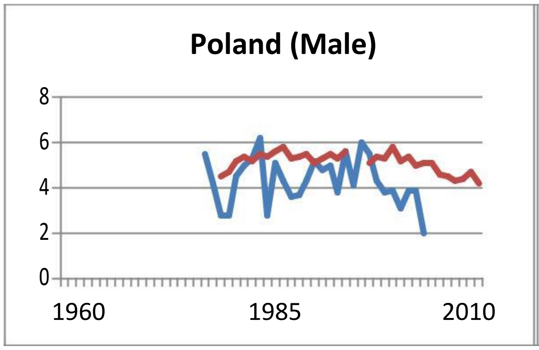

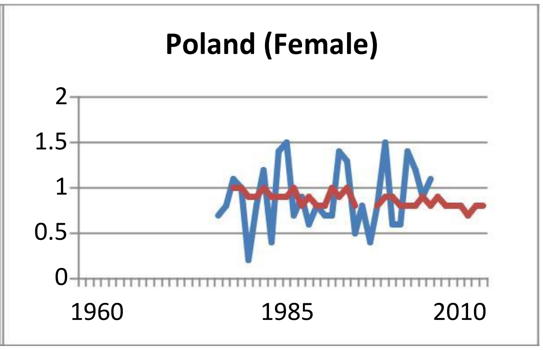

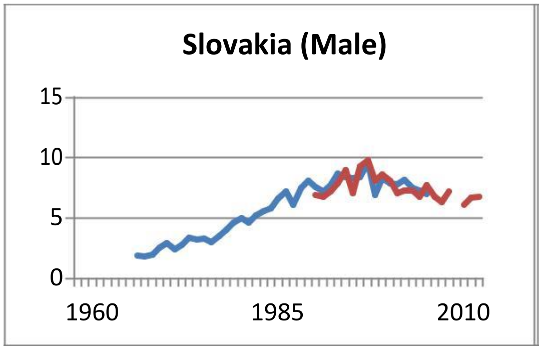

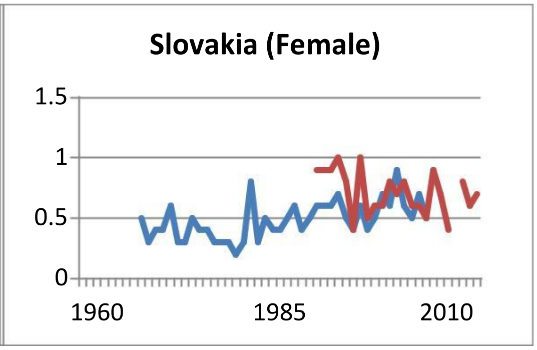

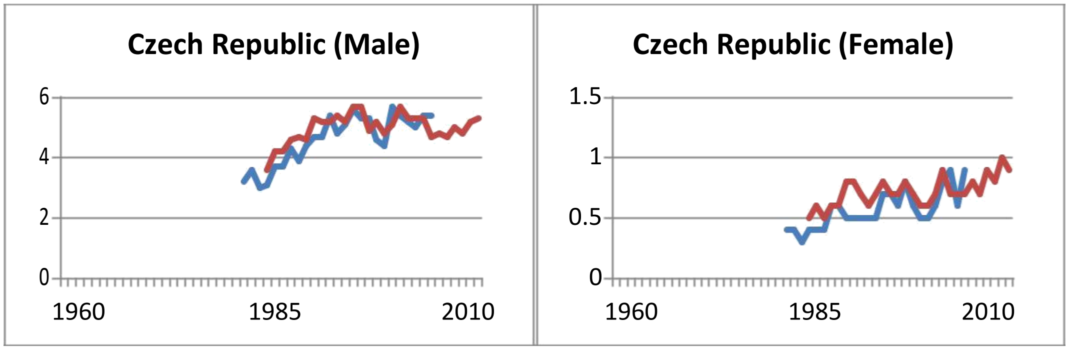
Czech Republic (Male)
6
4
2
0
1960
1985
2010
Czech Republic (Female)
1.5
1
0.5
0
1960
1985
2010
